# Machine learning approaches for assessing medication transfer to human breast milk

**DOI:** 10.1007/s10928-025-09972-9

**Published:** 2025-04-16

**Authors:** Zhongyuan Zhao, Peng Zou, Yuan Fang, Tong Si, Yanyan Li, Bofang Yi, Tao Zhang

**Affiliations:** 1 School of Pharmacy and Pharmaceutical Sciences, SUNY-Binghamton University, PO Box 6000, Binghamton, NY 13902 USA; 2 Ultragenyx Pharmaceutical Inc., 5000 Marina Blvd, Brisbane, CA 94005 USA; 3 Mathematics Department, Culver-Stockton College, One College Hill, Canton, MO 63435 USA; 4 Department of Mathematics and Statistics, SUNY-Binghamton University, PO Box 6000, Binghamton, NY 13902 USA

**Keywords:** Milk to plasma concentration ratio (M/P ratio), Machine learning, Breast milk, Pharmacokinetics

## Abstract

The human milk/plasma (M/P) drug concentration ratio is crucial in pharmacology, especially for breastfeeding mothers undergoing treatment. It determines the extent to which drugs ingested by the mother pass into breast milk, potentially affecting the infant. This study conducted a comprehensive evaluation of multiple machine learning algorithms to assess their effectiveness in predicting the M/P ratio. The dataset consists of 162 drugs and 11 predictor variables. M/P ratios were categorized into two groups of (0, 1) and (≥ 1), and a refined three-category system: (0, < 0.5), (0.5, < 1), and (≥ 1). The ML techniques utilized include K-Nearest Neighbors (KNN), Random Forest, Support Vector Machine (SVM), and Neural Networks. We implied the five-fold cross-validation to ensure the model’s robustness and Principal Component Analysis (PCA) was applied for data visualization. Bayesian Information Criterion (BIC) was used in the KNN model selection to balance complexity and explanatory power. In our study, KNN achieved average accuracies of 79% for the two-category system and 60% for the three-category. Random Forest models show 77 and 64% average accuracy, respectively. SVM achieved similar results with 78 and 67%, while Neural Networks have the overall best result among the other models with average accuracies of 82 and 76% accuracy. The study highlights the potential of machine learning (ML) techniques in predicting M/P ratios, offering valuable insights for risk assessment during drug development. These predictive models can serve as a valuable tool for estimating drug transfer into breast milk, helping to bridge knowledge gaps in drug safety for lactating individuals. Further validation and refinement by incorporating larger datasets can enhance their reliability and applicability. Advancing these techniques can support safer medication use and informed clinical decision-making for lactating individuals.

**Supplementary Information:**

The online version contains supplementary material available at 10.1007/s10928-025-09972-9.

## Introduction

Breastfeeding has been shown in multiple research studies to provide benefits for both the child and the mother [9, 28]. The Milk-to-Plasma (M/P) drug concentration ratio is a critical factor in clinical pharmacology, especially concerning the safety of medications for breastfeeding mothers [5, 15]. The M/P ratio provides an estimate of the extent to which a drug consumed by a lactating mother can pass into her breast milk and potentially affect the nursing infant [39]. With an increasing number of mothers requiring medication during lactation [4], there is a pressing need to accurately predict the transfer of drugs into human milk to ensure both the efficacy of the treatment for the mother and the safety of the infant. However, the direct measurement of drug concentrations in the milk and plasma of lactating women is often hindered by various challenges, including ethical and logistical issues related to recruiting lactating women and obtaining milk samples. As a result, only a small percentage of drugs have comprehensive human mammary excretion data available [35], posing a significant obstacle to risk assessment in breastfed infants [36]. So, developing models with machine learning methods to predict the M/P ratio can be helpful.

Various methodologies have been proposed to predict drug secretion into breast milk. Animal lactation studies are not very useful to predict human due to the large inter-species variability in protein and lipid contents of milk, difference in mammary gland anatomy, storage and release of milk, and drug transporters and metabolism [12]. Several Quantitative Structure Activity Relationship (QSAR) models have been developed to estimate drug transfer into human milk [1, 19, 21, 23]. These models typically start with dozens to hundreds of molecular descriptors generated by computational software based on compound structures. A subset of these descriptors is then selected using computational algorithms to optimize prediction accuracy. Additionally, a few in vitro cell-based assays have been reported to study drug transfer across mammary epithelium [7, 33, 38]. While these approaches have provided valuable insights, each has its limitations, and currently there is no reliable method to predict drug secretion into breast milk. Recent advancements in machine learning (ML) offer new opportunities to improve M/P ratio predictions by modeling complex, nonlinear interactions between drug properties and their transfer into milk.

In this study, we integrated the mechanistic understanding of drug secretion into breast milk with machine learning (ML) techniques to predict drug transfer into milk using a dataset of 162 drugs. Different from the QSAR approach that relies on many predicted molecular descriptors, we focused on a select set of eleven key parameters known to significantly impact milk-to-plasma (M/P) ratio. These parameters can be readily obtained from drug labels or standard experimental data, ensuring reliability, repeatability, and broad applicability. To predict the M/P ratio, we implemented multiple ML models, including K-Nearest Neighbors (KNN), Random Forest (RF), SVM, and Neural Networks (NN), and compared their predictions with observed data. The application of multiple ML approaches to categorizing the M/P ratio into distinct intervals allows for both dichotomized and more granular predictions. We improved the method further with a grid search of hyperparameters and explored the predictive power of these models across two different categorization schemes.

This study presents a novel, interpretable ML-based model grounded in drug secretion mechanisms, enhancing the accuracy and applicability of M/P ratio predictions to support safer medication use during lactation.

## Methods

### Data and sample

The dataset utilized in this study consists of 162 drugs with clinically observed M/P ratios, either directly collected from published literature or estimated based on the published drug exposure in the plasma and breastmilk (Supplementary Table 1). Only drugs that had ≥ 3 data points of paired plasma and milk concentrations available were selected. The M/P ratio was calculated as the ratio of milk AUC to plasma AUC [36]. Eleven variables were collected for each drug. These variables are drug unionized fractions at pH 7.0 (F_ni7.0_) and pH 7.4 (F_ni7.4_), drug fractions unbound in breast milk (f_um_), efflux ratios (ER) from Caco-2 cells, pKa, polar surface area (PSA), hydrogen bond donors (HBD), LogP, LogD7.4, molecular weight (MW), and elimination half-life (T_1/2_).The F_ni7.4_ and F_ni7.0_ values were predicted using SimCYP (Certara USA Inc., Princeton, NJ) prediction toolbox. The ER and fum values were obtained from literature [36]. The logD values were estimated based on the equations of calculating logD from logP for acids (logD = logP − log(1 + 10^(pH−pKa)^), bases (logD = logP − log(1 + 10^(pKa−pH)^), and neutral compounds (logD = LogP). The molecular properties (MW, HBD, logP, pKa, PSA) and PK parameters (T_1/2_) were obtained from drugbank website (https://go.drugbank.com/) or drug label (FDA).

For prediction purposes, the dataset was categorized into two M/P ratio ranges: [0, 1] and [1, 8], with the highest M/P ratio of 7.1. Additionally, we introduced a finer categorization system: [0, 0.5], [0.5, 1], and [1, 8] to enhance the granularity of the classification. All data were scaled prior to model application to improve performance and ensure consistent handling of drug properties. Data scaling ensures that variables with different ranges, such as drug molecular weight or efflux ratios, are treated equally by machine learning algorithms. In this study, we standardize each variable by subtracting the mean and dividing by the standard deviation. This method transforms the data so that each variable has a mean of 0 and a standard deviation of 1. This approach helps the model focus on the relationships between variables rather than being influenced by differences in their scales, which could otherwise lead to bias. By standardizing the variables, we improve the model’s ability to converge and enhance its predictive accuracy across a wide range of pharmacokinetic properties.

### Machine learning approaches for M/P ratio prediction

In this work, the features used in the training set included all 11 variables. In the later stages of this work, we also conducted feature importance ranking within each method, which will be introduced in subsequent sections. Model performance was evaluated using fivefold cross-validation. In each fold, 80% of the data was used for training and 20% for testing. Drugs were randomly assigned to these sets in Python to ensure an unbiased distribution of drugs across both sets. This random assignment helps avoid potential bias that could arise from systematic assignment, such as by drug name or specific drug properties. The prediction performance was measured by the mean accuracy, calculated as:

$$\textit{Mean Accuracy} = \sum\limits_{i = 1}^{5} {\frac{{\textit{Accuracy in i}{\mathrm{th}} \textit{fold}}}{\textit{Number of folds}}}$$

We employed several machine learning methods to predict the Milk/Plasma (M/P) ratio. K-Nearest Neighbors (KNN), as described by Guo et al. [13], classifies data points based on the majority class of their nearest neighbors using distance metrics, offering simplicity and effectiveness in classification tasks.

Support Vector Machine (SVM), from Hearst et al. [14] is well-suited for pharmaceutical data predictions due to its ability to separate data points with a hyperplane, efficiently handling both linear and non-linear relationships.

Neural Networks (NN), implemented using *TensorFlow* [32], model complex relationships through multiple layers of interconnected neurons, making them highly effective for non-linear data found in pharmaceutical applications.

Lastly, Random Forest (RF), introduced by Breiman [10] aggregates the results of multiple decision trees to reduce overfitting and improve prediction stability, making it a strong choice for complex datasets.

All methods were implemented using Python. KNN, SVM, and Random Forest were executed via the *Sklearn* [25] package, while Neural Networks were built using *TensorFlow* (TensorFlow [32]). The settings for each method will be introduced in the Result section of the grid search for each method.

## Results

### Selection of variables and PCA analysis

The dataset in this study includes 162 drugs, each characterized by 11 variables selected based on their mechanistic relevance to drug secretion into breast milk, focusing on key properties influencing drug passage through the mammary epithelium (Suppl. Table 1). Molecular properties that may facilitate drug transfer from plasma into breast milk include higher non-ionized drug fraction, lower plasma protein binding (higher unbound fraction), greater lipophilicity, smaller molecular weight (MW), lower polar surface area (PSA), and fewer hydrogen bond donors (HBD), all of which enhance passive diffusion [22, 34]. The efflux ratio (ER) serves as an indicator of drug permeability and the involvement of active efflux transporters. The breast cancer resistance protein (BCRP) has been reported to play an important role in drug accumulation into breast milk [2, 12]. Additionally, elimination half-life (T_1/2_) is a key pharmacokinetic (PK) parameter that reflects the drug’s duration in systemic circulation. A longer T_1/2_ suggests prolonged drug presence in milk, potentially increasing infant exposure. Collectively, these variables influence drug secretion into breast milk, though some may have overlapping roles in the transfer process.

**Fig. 1:**
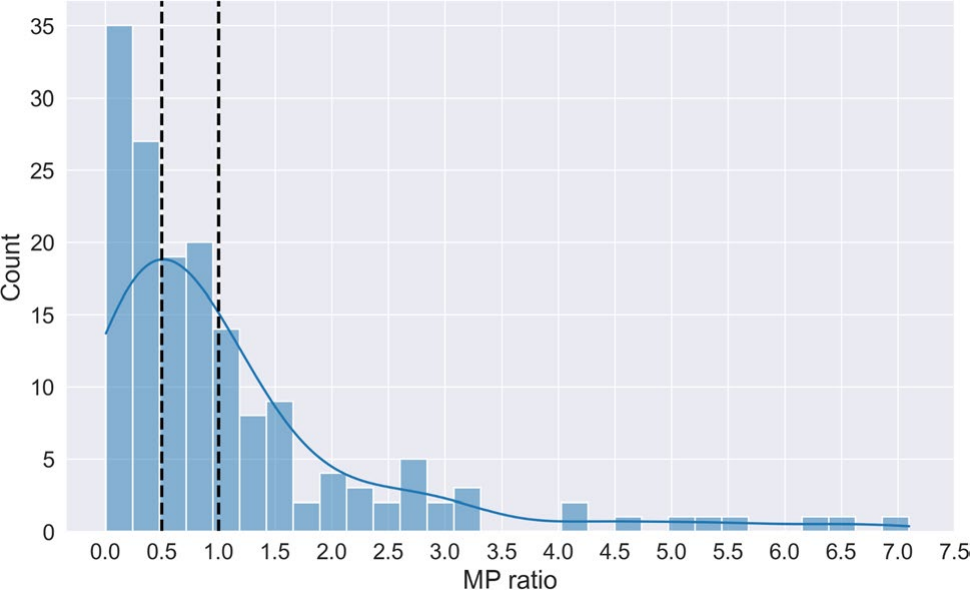
Distribution of observed M/P ratio for the compiled dataset of 162 drugs. The two dash lines define the three categories classification system with M/P ratio range of [0, 0.5], [0. 5,1], and [1, 8]

The observed M/P ratios range from 0.005 to 7.1, with the majority of values clustering around 1 (Fig. 1). For a two categories classification, the ranges [0, 1] and [1, 8] were used, where 1 indicates equal concentrations in plasma and breast milk. In the three categories classification system, the ranges [0, 0.5], [0.5, 1], and [1, 8] represent low, moderate, and high breast milk concentrations relative to plasma concentrations. With this three categories categorization, each category contains approximately 40–60 drugs.

We initially performed a Principal Component Analysis (PCA) on the scaled data, focusing on Principal Components 1 and 2 as representative dimensions (95% total variation explained) for exploratory visualization (Supplement Fig. 1). PCA helps to reveal potential underlying patterns in the data by transforming the original features into a smaller set of uncorrelated components that may capture the majority of the variance [18]. Given that our dataset has only 11 features, dimensionality reduction is not a primary concern, and PCA was used solely for data exploration rather than as a preprocessing step for modeling.

### K-Nearest neighborhood

**Fig. 2:**
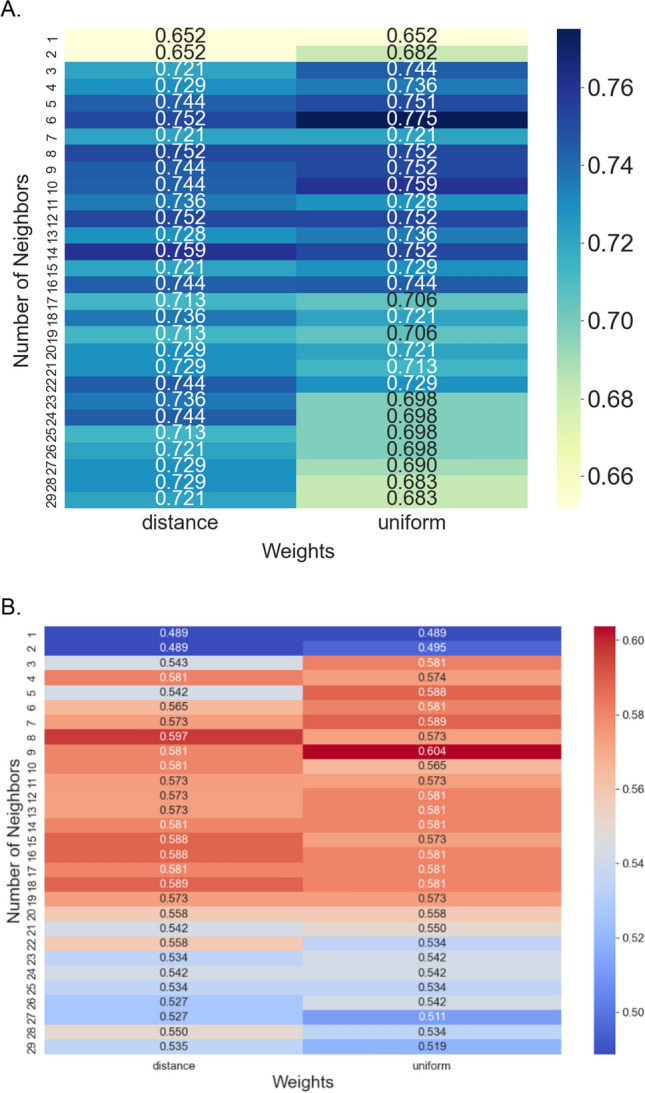
Prediction results of the K-Nearest Neighbors (KNN) models. **A** Heatmap of two categories KNN model average accuracy by number of neighbors and weight type. **B** Heatmap of three categories KNN model average accuracy by number of neighbors and weight type

To optimize the KNN model for two-category predictions, we employed a grid search with fivefold cross-validation [17] to identify the optimal hyperparameters. The grid search results indicated that the best combination of hyperparameters included the use of Euclidean distance among predicting variables, with 6 neighbors and uniform weights. The cross-validation process yielded an average accuracy score of 78% (Fig. 2A) with a 0.04 standard deviation and mean F-1 score of 0.7.

For the KNN model applied to three categories of the MP ratio, we initially set the number of neighbors to 5, used Euclidean distance, and applied uniform weights, which resulted in an accuracy of 67%. To enhance the model’s generalization, we again employed a grid search with fivefold cross-validation. The optimized parameters recommended by this process were Euclidean distance, 9 neighbors, and uniform weights (Fig. 2B). However, this adjustment led to a decrease in average accuracy to 60% with a standard deviation of 0.06 and mean F-1 score of 0.57.

### Random forest

**Fig. 3:**
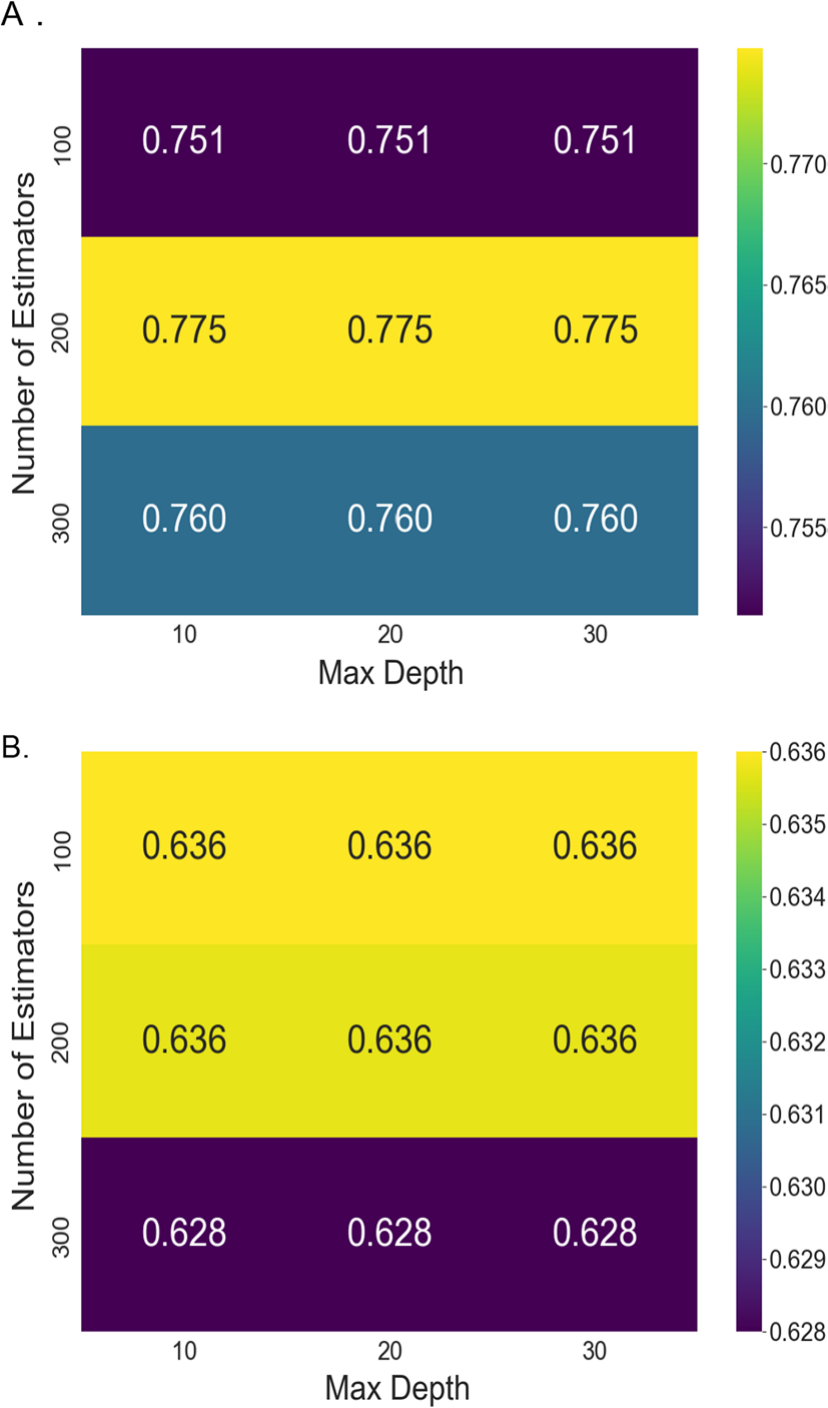
Prediction results of random forest method. **A** Grid search results heatmap for two categories random forest with average accuracy. **B** Grid search results heatmap for three categories random forest with average accuracy

For the random forest method applied to two categories of the MP ratio, we initially set the number of trees to 100 and utilized the default parameters provided by the *Sklearn* package in Python. This approach resulted in an accuracy of 73%. Subsequently, we conducted a grid search with fivefold cross-validation to optimize the hyperparameters. The grid search identified the best parameters as follows: any maximum depth, a minimum sample leaf of 1, a minimum sample split of 10, and the number of estimators (trees) set to 200. This configuration improved the average accuracy to 78% (Fig. 3A) with a standard deviation of 0.07 and mean F-1 score of 0.69, which is a slight enhancement over the default settings.

For the random forest method applied to three categories of the MP ratio, we again started with 100 trees and the default settings. The initial accuracy achieved was 60%. The grid search returned a similar configuration, with the primary difference being the number of estimators, which remained at 100. In the subsequent cross-validation, this setup yielded an accuracy of 64% (Fig. 3B) with the same standard deviation of 0.07 and mean F-1 score of 0.62, demonstrating a modest improvement.

We further explore the importance of features in both the two categories (Supplement Fig. 2A) and three categories (Supplement Fig. 2B); Notably, ER is the most influential predictor across both models, indicating its strong impact on classification performance. Comparing the results from the two categories and three categories scenario, suggest that while ER consistently drives the primary variation in predictions, the importance of other features can shift depending on the complexity of the classification problem.

### Support vector machine

**Fig. 4:**
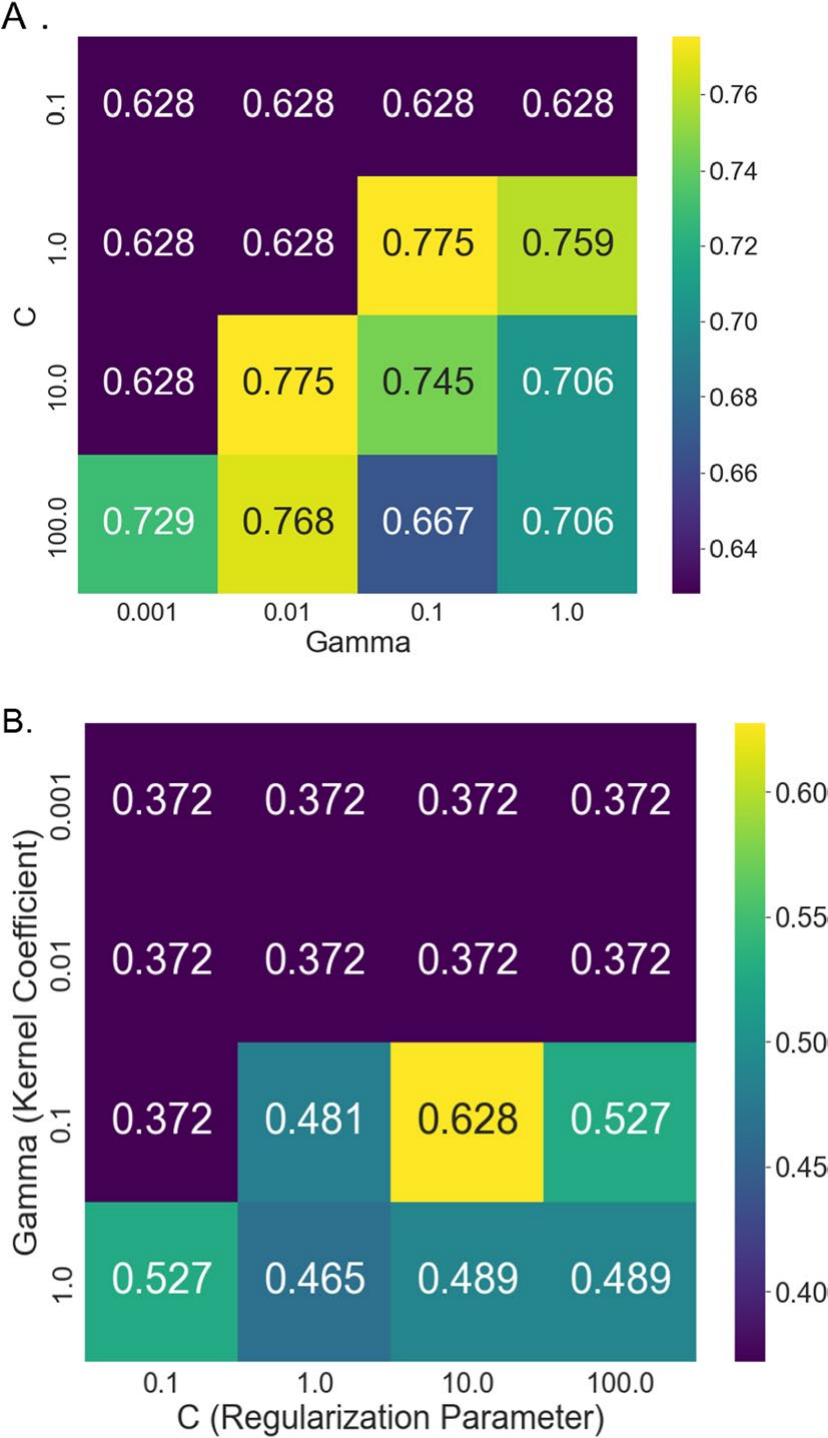
Prediction results of Support Vector Machine (SVM) method. **A** Grid search results heatmap for two categories SVM with average accuracy. **B** Grid search results heatmap for three categories SVM with average accuracy

For the SVM method applied to two categories of the MP ratio, we initially set the kernel to the radial basis function (RBF), achieving an accuracy of 76%. Subsequently, we performed a grid search to optimize the regularization parameter C, the kernel coefficient γ, and the type of kernel. The grid search with fivefold cross-validation identified the optimal parameters as C = 10, γ = 0.01, and confirmed that the RBF remained the best kernel choice. This resulted in an improved average accuracy of 78% (Fig. 4A) with a standard deviation of 0.03 and mean F-1 score of 0.71.

For the SVM applied to three categories of the MP ratio, the initial settings yielded an accuracy of 67%. Another grid search with fivefold cross-validation was conducted to refine these parameters. The optimal configuration was found to be the same regularization parameter of C = 10, a kernel coefficient of γ = 0.1, but with the kernel type changing to a polynomial kernel. Although the accuracy slightly decreased to 63% (Fig. 4B) with a standard deviation of 0.02 and mean F-1 score of 0.60, this setting offered better generalization.

We further explore the feature importance of two categories (Supplement Fig. 3A) and three categories (Supplement Fig. 3B) in the SVM model. In the two categories scenario, f_um_ emerges as a top predictor, followed closely by F_ni7.0_ and F_ni7.4_, suggesting that these features are most critical for distinguishing between the two classes. In contrast, the three categories classification highlights F_ni7.0_ as the most influential feature, with f_um_, F_ni7.4_, and LogP also contributing notably. This shift in the most important features underlines how expanding the classification from two categories to three changes the model’s reliance on specific properties, providing insight into the varying drivers of class separation.

### Basic neural networks

**Fig. 5:**
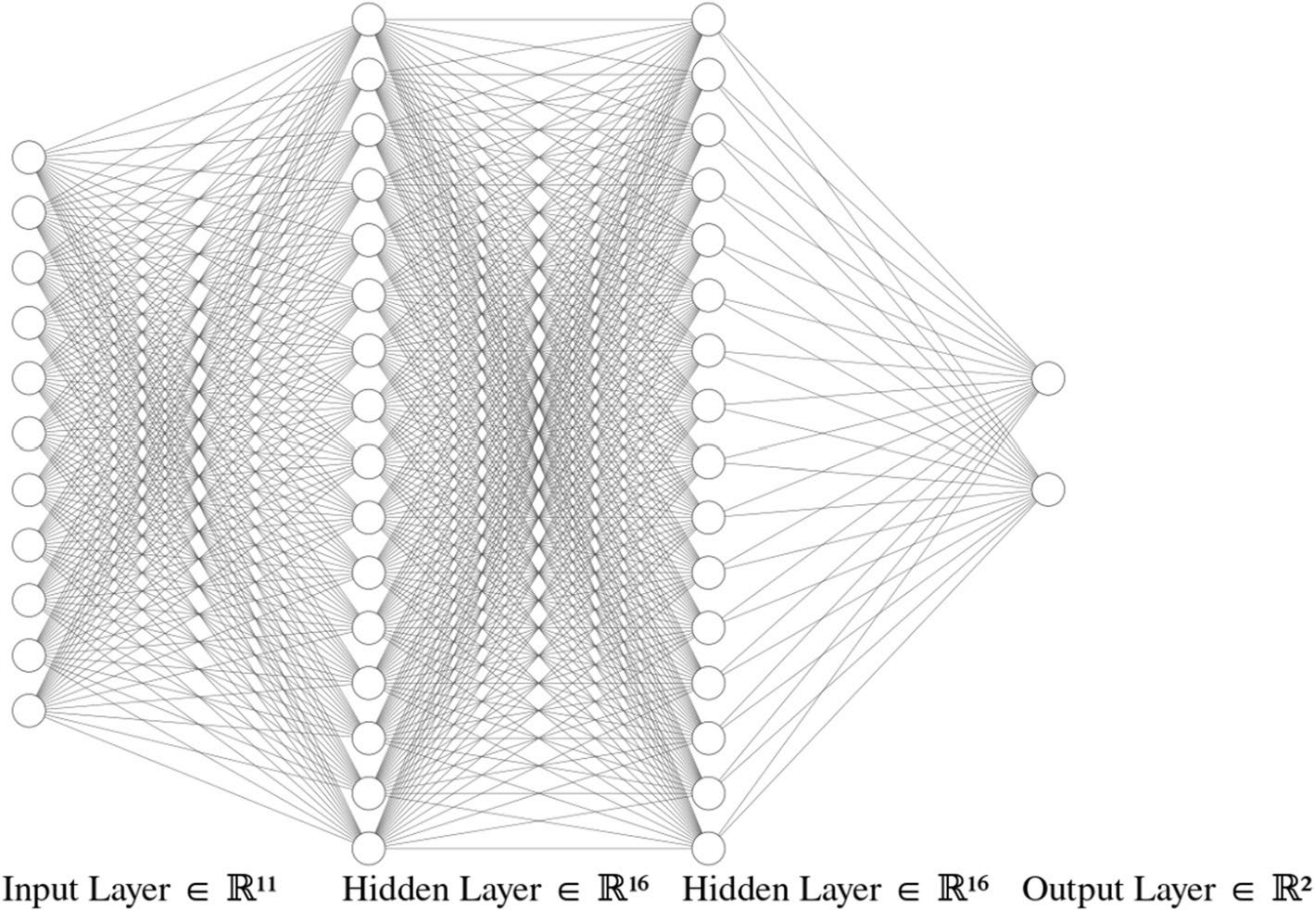
Neural Network structure for two categorial predictions. The Input Layer (far left) contains 11 nodes, each representing a different characteristic or feature of the drug. The Hidden Layers (middle) contain 16 nodes each, which help the model learn complex patterns by connecting inputs to outputs through several processing steps. Finally, the Output Layer (far right) has 2 nodes, each representing one of the two categories of predicted M/P ratios. Lines between nodes represent connections that pass information from one layer to the next

For the neural network method applied to two categories of the MP ratio, we initially configured the model with two hidden layers, each containing 16 neurons, total of 32 neurons (Fig. 5). The *ReLU* activation function was used in the hidden layers, while the *softmax* activation function was employed in the output layer. [29] The Adam optimizer and Sparse Categorical Cross entropy were chosen as the optimization and loss functions, respectively. We set the number of epochs to 50 and the batch size to 10, which resulted in an accuracy of 79%.

**Fig. 6:**
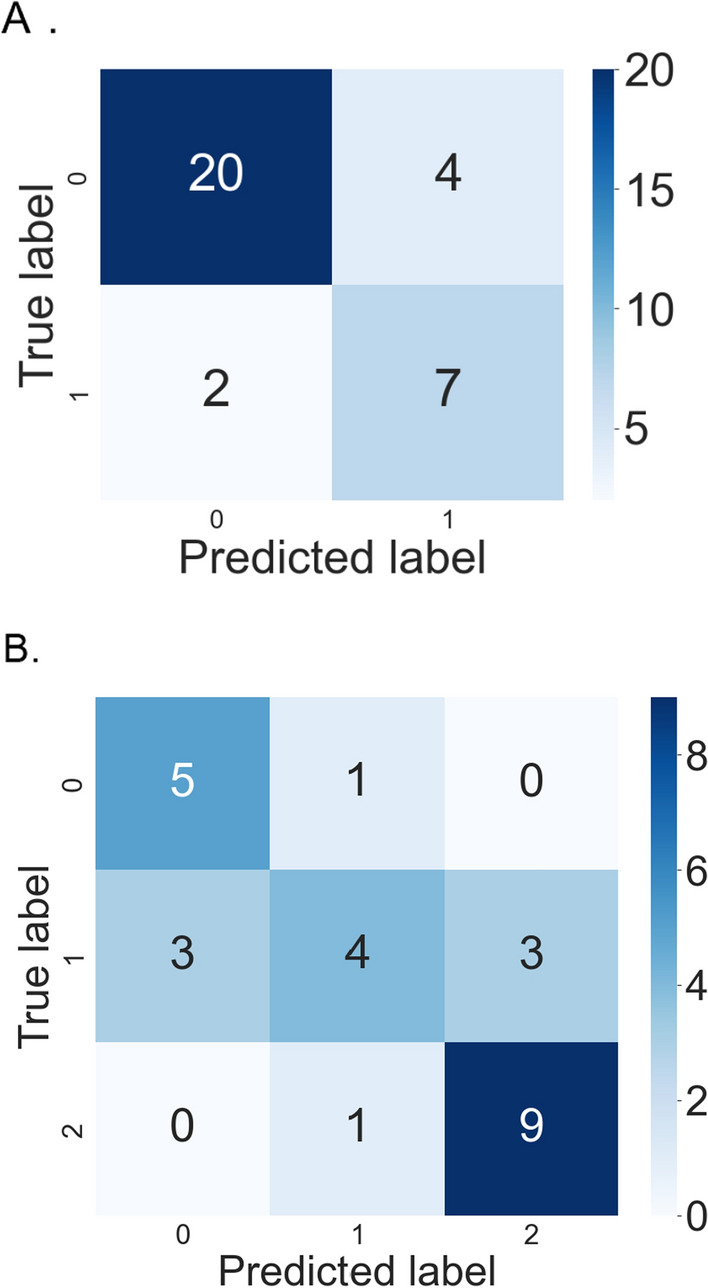
Prediction results of Neural Network method. **A** Heatmap for an example of two categories neural network. **B** Heatmap for an example of three categories neural network

Subsequently, we tuned the model by reducing the neuron activations in the hidden layers to 0.5 [30], which will improve the neural network by Srivastava et al. [30], and changing the activation function in the output layer to sigmoid. Additionally, we implemented an early stopping mechanism to monitor the loss of accuracy on the testing set, halting the training process if accuracy began to decrease. This can reduce the chance of having an overfitting model to increase the performance of the model. These modifications increased prediction accuracy from 76 to 82% (Fig. 6A) with a 0.09 standard deviation and mean F-1 score of 0.82 (Table 1).

**Table 1:** Summary of prediction accuracy from all methods

Classification	Method	Average accuracy (%)	SD of accuracy	Average F-1 score
Two categories	KNN	78	0.04	0.70
	RF	78	0.07	0.69
	SVM	78	0.03	0.71
	NN	82	0.09	0.82
Three categories	KNN	60	0.06	0.57
	RF	64	0.07	0.62
	SVM	63	0.02	0.60
	NN	68	0.12	0.61

For the neural network applied to three categories of the MP ratio, we initially used the same configuration as for the two categories, achieving an accuracy of 64%. We then modified the model by increasing the number of neurons in the first hidden layer to 64 and in the second hidden layer to 32. This adjustment resulted in an accuracy of 76%, which was the highest accuracy achieved for the three-category predictions compared to other methods applied earlier. However, after we run a cross-validation, it returns an average accuracy of 68% (Fig. 6B) with a standard deviation of 0.12 and mean F-1 score of 0.61, which is relatively high.

We also explored the importance of features for the Neural Network model in both two categories (Supplement Fig. 4A) and three categories (Supplement Fig. 4B) classification settings. PSA consistently emerges as the top contributor, which indicates it is important in the model’s decision-making process across both classification schemes. In the two categorial analysis, f_um_ and F_ni7.0_ also rank highly. Meanwhile, in the three categories classification, ER and f_um_ rank highly, reflecting the relative impact of certain feature shifts when the classification task becomes more difficult.

### Performance comparison

Among the evaluated models, Neural Networks achieved the highest average accuracy, reaching 82% with a 0.09 standard deviation for the two-category M/P ratio classification and 68% with a standard deviation of 0.12 for the three-category classification, but for the three-category classification, Neural Networks returned a high standard deviation. K-Nearest Neighbors also demonstrated acceptable performance, particularly in the two-category classification, where it achieved an average accuracy of 78% with a 0.04 standard deviation. However, its performance slightly declined to 60% with a standard deviation of 0.06 when extended to three categories. Although Support Vector Machines are widely applied in the prediction of pharmaceutical properties [8], in our case, it showed slightly lower performance, with an average accuracy of 78% with a standard deviation of 0.03 and 63% with a standard deviation of 0.02 for two and three categories, respectively. Random Forest achieved an average accuracy of 78% with a standard deviation of 0.02 and 64% with a standard deviation of 0.02 for two and three categories, respectively. The improved accuracy after hyperparameter tuning indicates the importance of model configuration in enhancing performance.

**Fig. 7:**
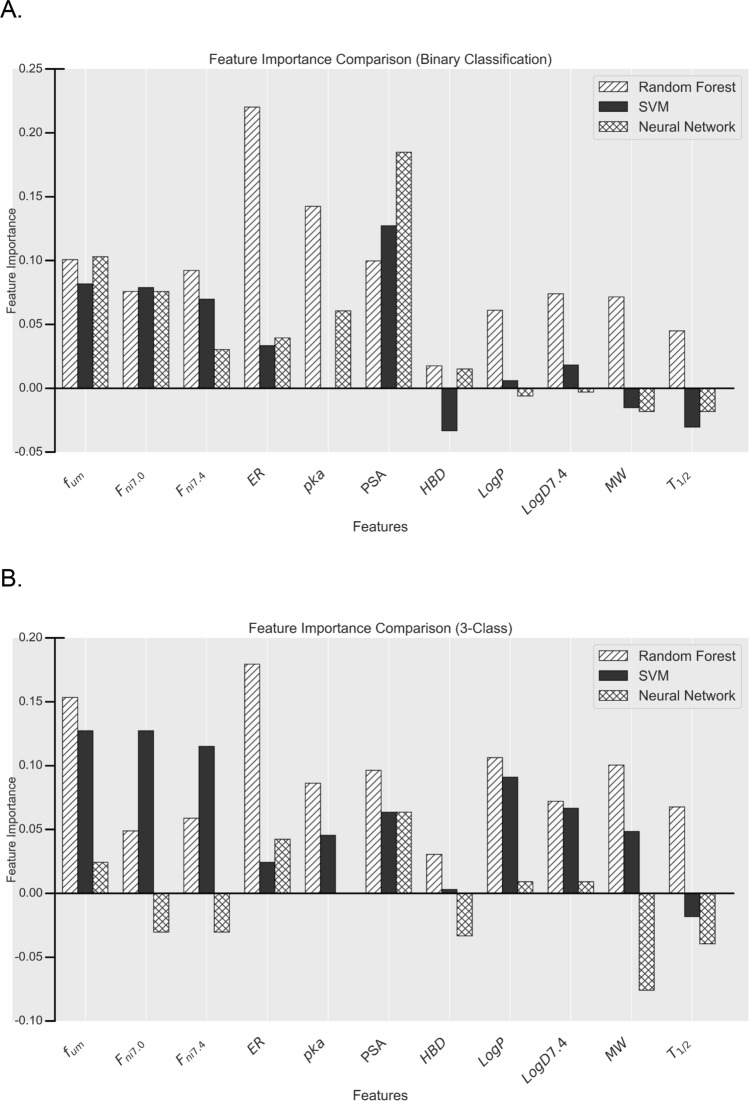
Feature importance comparison of Random Forest, SVM, and Neural Network. **A** The bar plot of feature importance comparison of RF, SVM, and NN with two categories classification system. **B** The bar plot of feature importance comparison of RF, SVM, and NN with three categories classification system

Since KNN does not have feature importance, we compare the other three models side by side, and several patterns emerge for the two categories classification (Fig. 7A). Notably, PSA appears as a dominant predictor in both the SVM and Neural Network models, while for the Random Forest model, ER holds the top spot. This suggests that each algorithm captures different aspects of the data structure. For instance, Random Forest may be more sensitive to certain interaction patterns captured by ER, while SVM and the Neural Network place greater emphasis on PSA and related physicochemical properties such as f_um_ and F_in7.0_. Additionally, features like pka, LogP, and F_in7.4_ vary in their relative importance across the models, indicating that they contribute to classification accuracy but to differing degrees. In some cases, features like T_1/2_, MW, and HBD show lower or even negative importance, suggesting that deleting these features either does not harm performance or can unexpectedly improve it, possibly due to correlations with other predictors.

For the three categories classification, each model highlights a different feature as the top predictor (Fig. 7B). For instance, ER emerges as the most critical feature in the Random Forest model, while F_in7.0_ and f_um_ tops the list for the SVM, and PSA is most influential for the Neural Network. Despite these differences, features like LogP and f_um_ consistently appear among the higher-ranked predictors, suggesting that they also play a significant role in classification. Meanwhile, features such as T_1/2_ or HBD often exhibit lower or even slightly negative importance, indicating that omitting them may not degrade model performance, or might even improve it, due to correlations with other variables.

Overall, this comparison shows how each algorithm’s unique learning mechanism emphasizes different properties of the dataset, reinforcing the value of testing multiple models to gain a well-rounded understanding of the features that most strongly influence two categories and three categories classification.

## Discussion

In this study, we investigated the accuracy of various machine learning algorithms in predicting the M/P ratio of 162 drugs using 11 drug properties. The primary objective was to predict the M/P ratios using various ML tools, including KNN, SVM, RF, and NN. Our findings reveal that Neural Networks consistently outperformed the other models in both two-category and three-category classification schemes, highlighting their robustness in handling complex pharmaceutical science data. This is particularly significant as Neural Networks are widely recognized for image processing [26]; however, their superior performance in pharmacokinetic prediction highlights their adaptability and potential in modeling complex biological processes. In recent years, advanced neural network models have shown remarkable improvements in various domains [20], and our findings further extend their applicability to pharmacokinetic predictions.

For the accuracy of the Neural Network method. This superior performance can be attributed to the inherent capability of neural networks to capture non-linear relationships and interactions among the input features [3], which are often present in pharmacokinetic data. The implementation of early stopping and the adjustment of activation functions further enhanced the model’s generalization ability, preventing overfitting and ensuring better performance on unseen data [37]. However, the high standard deviation in three-category prediction also indicates the limitation of this model due to the small dataset within each category. And the performance of KNN gives us more information about the data. The decrease in accuracy in prediction may stem from the increased complexity and finer granularity of the classification task, which challenges KNN’s reliance on local proximity measures. Since the difference between each variable in pharmacokinetic data varies in values can be large. Despite this, KNN remains a viable option due to its simplicity and interpretability. The optimization of hyperparameters through grid search improved the SVM’s performance, although the gains were relatively modest compared to Neural Networks. The transition from a radial basis function to a polynomial kernel in the three-category classification resulted in a slight decrease in accuracy, suggesting that the chosen kernel may not have been optimal for the finer classification task. For Random Forest, we got improvement during hyperparameter tuning. However, similar to all the other methods, the performance decreased with additional categories, reflecting the challenges posed by more complex classification schemes.

The categorization of the M/P ratio into two and three ranges revealed that increasing the number of categories generally led to a decrease in model accuracy across all machine learning methods. This trend underscores the added complexity and the potential for overlap between categories, which can hinder the model’s ability to accurately distinguish between classes. Specifically, while the two-category classification provided clearer separation, the three-category scheme introduced finer distinctions that some models struggled to capture effectively.

The application of Principal Component Analysis (PCA) in the KNN model demonstrated limited benefits. Although dimensionality reduction is typically employed to enhance model performance by eliminating multicollinearity and reducing noise [6], in this case, PCA did not significantly improve accuracy and even led to a slight decrease in the two-category classification. This outcome suggests that the original feature set was already sufficiently informative, and further reduction may have inadvertently removed pertinent information necessary for accurate predictions.

One of the strengths of this study is the comprehensive evaluation of multiple machine learning (ML) algorithms, providing a comparative analysis of their performance in predicting the M/P ratio. The use of hyperparameter optimization and techniques such as early stopping in Neural Networks further strengthens the predictive accuracy of the methods.

However, the study also has several limitations. The relatively small dataset of 162 drugs may limit the generalizability of the findings and increase the risk of overfitting [27], especially for complex models like Neural Networks. Concerns have been raised in the literature about the lack of a holdout test set potentially inflating training accuracy and leading to overfitting [31]. Given the limitations of the size of our dataset, the absence of a separate validation set in our study may increase the risk of overfitting and limit the generalizability of our findings. Additionally, the study focused solely on classification accuracy as the performance metric, without considering other important metrics such as precision, recall, or the area under the Receiver Operating Characteristic (ROC) curve, which could provide a more nuanced understanding of model performance. Furthermore, the variable selection process [24] mentioned in the methodology was not elaborated upon in the results, leaving a gap in understanding how feature importance and selection influenced the models’ performance. Future studies should explore the impact of different feature selection techniques and their integration with various machine learning algorithms.

The findings from this study indicate that Neural Networks are particularly effective for predicting the M/P ratio of drugs, especially when navigating the complexities of non-linear relationships among variables. This result supports the emerging research suggesting the benefit of ML for drug development and personalized medicine, where the accurate prediction of pharmacokinetic parameters is critical. Neural Networks’ ability to model intricate, non-linear interactions within data positions them as a powerful tool for understanding complex biological systems. This effectiveness is further supported by their successful application in modeling biological networks and processes, as evidenced by previous research. These models have demonstrated their value in uncovering hidden patterns within regulatory networks and signaling pathways—key components in the progression of diseases such as cancer [11, 16]. However, like other models, Neural Networks are not immune to the limitations caused by small datasets. Insufficient data can lead to higher prediction errors and increased variability, as reflected in larger standard deviations in our three-category prediction.

Future research should aim to validate these findings with larger and more diverse datasets to enhance the robustness and generalizability of the models. However, accessing more datasets can be a challenging problem. Exploring ensemble methods that combine the strengths of multiple algorithms could potentially yield even higher accuracy and reliability. Incorporating additional features, such as genetic or metabolic data, might also improve prediction performance and provide deeper insights into the factors influencing the M/P ratio. Moreover, expanding the evaluation to include other performance metrics and conducting a more detailed analysis of feature importance would offer a more comprehensive understanding of the models’ capabilities and limitations. Finally, investigating the application of advanced neural network architectures, such as convolutional or recurrent neural networks, could further enhance predictive performance and uncover intricate patterns within the data.

## Conclusion

Machine learning (ML) has rapidly advanced across various fields of research, including pharmaceutical sciences. In this study, we conducted a comprehensive evaluation of multiple ML approaches for predicting the Milk-to-Plasma (M/P) drug concentration ratio, achieving accuracy exceeding 80% in the best-performing models. These findings demonstrate that ML techniques are promising tools for advancing research in this area, providing valuable insights for risk assessment of compounds during early-stage development. Future efforts should focus on validating these results with larger and more diverse datasets to improve the robustness and generalizability of the models, paving the way for broader applications in drug safety during lactation.

## Supplementary Information

Below is the link to the electronic supplementary material.

Supplementary file1 (DOCX 2632 KB)

## Data Availability

The data that support the findings of this study are available from the corresponding author upon reasonable request.

## Declarations

### Generative AI in scientific writing

During the preparation of this work the author(s) used Grammarly in order to correct the spelling and grammar mistakes. After using this tool/service, the author(s) reviewed and edited the content as needed and take(s) full responsibility for the content of the publication.
