# Supplementary material for: Machine learning approaches for assessing medication transfer to human breast milk
Source: J Pharmacokinet Pharmacodyn. 2025 Apr 16;52(3):25. doi: 10.1007/s10928-025-09972-9 (PMC13249712; doi:10.1007/s10928-025-09972-9)

**Supplementary Table 1: Summary of physicochemical properties, PK parameters, and M/P ratios of 162 drugs**

| Drug | f_um_^a^ | F_ni7.0_^b^ | F_ni7.4_^b^ | ER^a^ | M/P_Obs_^c^ | pka^d^ | PSA^d^ | HBD^d^ | LogP^d,e^ | LogD7.4^e^ | MW^d^ | T_1/2_^d^ |
| --- | --- | --- | --- | --- | --- | --- | --- | --- | --- | --- | --- | --- |
| acebutolol | 0.945 | 0.00268 | 0.00671 | 1.37 | 7.1003 | 9.57 | 87.7 | 3 | 1.71 | -0.42 | 336.4 | 3.5 |
| acetaminophen | 0.84 | 0.997 | 0.991 | 1.37 | 1.2406 | 9.46 | 49.3 | 2 | 0.46 | 0.39 | 151.2 | 2.5 |
| acetazolamide | 1.007 | 0.613 | 0.387 | 0.962 | 0.3 | 7.2 | 152 | 2 | -0.3 | -0.71 | 222.3 | 4 |
| acyclovir | 1.09 | 0.994 | 0.986 | 2.6561 | 1.58011 | 9.25 | 114.8 | 4 | -1.56 | -1.66 | 225.2 | 2.9 |
| alprazolam | 0.74 | 0.988 | 0.995 | 0.962 | 0.46 | 5.08 | 43.1 | 0 | 2.12 | 2.12 | 308.8 | 11.2 |
| aminosalycylic acid | 0.987 | 7.41E-09 | 1.18E-09 | 1.70015 | 0.13 | 3.68 | 83.6 | 3 | 0.89 | -2.65 | 153.1 | 0.5 |
| amitriptyline | 0.75 | 0.00173 | 0.00435 | 1.78 | 1.11019 | 9.76 | 3.2 | 0 | 5 | 3.03 | 277.4 | 19 |
| amoxapine | 0.82 | 0.0146 | 0.0358 | 0.962 | 0.21 | 8.83 | 36.9 | 1 | 3.4 | 1.95 | 313.8 | 8 |
| amphetamine | 0.941 | 0.00125 | 0.00315 | 0.5202 | 4.9808 | 9.9 | 26 | 1 | 1.8 | -0.58 | 135.2 | 10 |
| atenolol | 0.96 | 0.0025 | 0.00627 | 1.16 | 3.1206 | 9.6 | 84.6 | 4 | 0.5 | -1.73 | 266.3 | 7.5 |
| aztreonam | 1.215 | 2.90E-08 | 1.80E-08 | 0.9232 | 0.00724 | -0.7 | 206 | 3 | -2.069 | -3.07 | 435.4 | 1.7 |
| baclofen | 0.968 | 1.79E-06 | 1.79E-06 | 0.77025 | 0.82026 | 3.87 | 63.3 | 2 | 1.3 | -0.96 | 213.7 | 4 |
| benznidazole | 0.988 | 1 | 1 | 1.78 | 0.52028 | 13.6 | 92.7 | 1 | 0.9 | 0.9 | 260.2 | 12.1 |
| bepridil | 0.662 | 0.00687 | 0.0171 | 0.962 | 0.33016 | 9.16 | 15.7 | 0 | 5.2 | 3.43 | 366.5 | 33 |
| budesonide | 0.28 | 1 | 1 | 1.37 | 0.4606 | 11.8 | 93.1 | 2 | 1.9 | 1.9 | 430.5 | 4 |
| bupivacaine | 0.798 | 0.0736 | 0.166 | 1.37 | 0.34032 | 8.1 | 32.3 | 1 | 3.4 | 2.62 | 288.4 | 2.7 |
| bupropion | 0.42 | 0.0568 | 0.132 | 0.4972 | 2.8006 | 7.9 | 29.1 | 1 | 3.6 | 2.72 | 239.7 | 3.5 |
| caffeine | 1.092 | 1 | 1 | 1.046 | 0.7106 | 0.7 | 58.4 | 0 | -0.5 | -0.5 | 194.2 | 5 |
| captopril | 1.014 | 5.00E-04 | 0.000199 | 0.51634 | 0.0356 | 3.7 | 96.4 | 2 | 0.6 | -2.77 | 217.3 | 2 |
| carbamazepine | 0.884 | 1 | 1 | 0.5202 | 0.46535 | 15.96 | 46.3 | 1 | 2.5 | 2.5 | 236.3 | 35 |
| cefpodoxime proxetil | 1.052 | 1 | 1 | 0.962 | 0.08016 | 14 | 156.4 | 3 | 0.05 | -3.73 | 427.5 | 2.4 |
| cefprozil | 1.012 | 9.18E-05 | 6.52E-05 | 0.962 | 0.5306 | 2.92 | 158.3 | 5 | 0.6 | -2.99 | 389.4 | 1.5 |
| cephalothin | 0.93 | 0.000426 | 0.00017 | 1.25536 | 0.1506 | 3.63 | 166.6 | 2 | -0.2 | -3.54 | 396.4 | 0.5 |
| chloramphenicol | 1 | 0.755 | 0.552 | 1.0952 | 0.5006 | 7.49 | 115.4 | 3 | 0.7 | 0.7 | 323.1 | 5.1 |
| chloroquine | 0.792 | 0.000794 | 0.00199 | 1.37 | 6.60038 | 10.1 | 28.2 | 1 | 4.693 | 1.59 | 319.9 | 1200 |
| chlorprothixene | 0.723 | 0.038 | 0.091 | 0.962 | 1.48039 | 8.4 | 3.2 | 0 | 5.18 | 3.57 | 315.9 | 10 |
| cimetidine | 0.89 | 0.613 | 0.799 | 3.226 | 4.1806 | 6.8 | 114.2 | 3 | 1 | 0.89 | 252.3 | 2.5 |
| ciprofloxacin | 0.966 | 0.00224 | 0.00233 | 2.764 | 1.4954 | 6.09 | 72.9 | 2 | 1.308 | -0.29 | 331.3 | 5 |
| citalopram | 0.846 | 0.00166 | 0.00415 | 1.78 | 1.43 | 9.78 | 36.3 | 0 | 3.2 | 1.15 | 324.4 | 36 |
| clarithromycin | 0.89 | 0.0101 | 0.0251 | 1.37 | 0.2206 | 8.99 | 182.9 | 4 | 1.7 | 0.68 | 748 | 3.5 |
| clindamycin | 0.99 | 0.22 | 0.414 | 0.962 | 0.94016 | 7.55 | 127.6 | 4 | 1.6 | 1.21 | 425 | 3 |
| codeine | 0.978 | 0.0594 | 0.137 | 0.8702 | 2.1206 | 8.2 | 41.9 | 1 | 1.19 | -0.6 | 299.4 | 2.9 |
| cotinine | 1.082 | 0.994 | 0.998 | 0.962 | 0.78044 | 4.79 | 33.2 | 0 | -0.3 | -0.3 | 176.2 | 16 |
| dapsone | 0.973 | 1 | 1 | 1.00045 | 0.34046 | 14 | 86.2 | 2 | 0.936 | 0.99 | 248.3 | 30 |
| desipramine | 0.81 | 4.00E-04 | 0.000999 | 1.6 | 1.45047 | 10.4 | 15.3 | 1 | 3.972 | 1.27 | 266.4 | 33 |
| desmethyldoxepin | 0.822 | 0.00034 | 0.00085 | 0.962 | 1.27548 | 10.47 | 21.3 | 1 | 3.8 | 1.06 | 265.3 | 16.8 |
| diazepam | 0.334 | 1 | 1 | 0.913 | 0.20049 | 3.3 | 32.7 | 0 | 2.82 | 2.8 | 284.7 | 48 |
| digoxin | 1.06 | 0.585 | 0.36 | 1.37 | 1.2106 | 7.15 | 203.1 | 6 | 2.2 | 2.2 | 780.9 | 40 |
| diltiazem | 0.53 | 0.062 | 0.142 | 1.37 | 0.9906 | 8.18 | 84.4 | 0 | 2.8 | 1.96 | 414.5 | 3.8 |
| disopyramide | 0.98 | 4.00E-04 | 0.000999 | 0.962 | 0.9 | 10.4 | 59.2 | 2 | 3.5 | 0.6 | 339.5 | 7 |
| dothiepin | 0.786 | 0.0017 | 0.0043 | 0.962 | 1.70053 | 9.76 | 28.5 | 0 | 4.5 | 2.22 | 295.4 | 19 |
| doxazosin | 0.792 | 0.751 | 0.883 | 0.962 | 0.10054 | 6.52 | 112 | 1 | 2.5 | 2.45 | 451.5 | 11 |
| doxepin | 0.801 | 0.011 | 0.027 | 1.53055 | 1.37048 | 8.96 | 12.5 | 0 | 4.3 | 2.74 | 279.4 | 15 |
| doxorubicin | 0.894 | 0.059 | 0.136 | 7.626 | 1.19 | 8.2 | 206.1 | 6 | 2.289 | -1.47 | 543.5 | 34 |
| doxycycline | 1.064 | 1 | 1 | 0.962 | 0.3408 | 3.09 | 182 | 6 | -0.7 | -0.7 | 444.4 | 19 |
| escitalopram | 0.846 | 0.00166 | 0.00415 | 0.90122 | 2.2106 | 9.78 | 36.3 | 0 | 3.5 | 1.15 | 324.4 | 30 |
| ethanol | 1.067 | 1 | 1 | 0.962 | 0.9006 | 16 | 20.2 | 1 | -0.1 | -0.1 | 46.1 | 4.5 |
| ethosuximide | 1.032 | 1 | 1 | 0.962 | 0.80061 | 10.73 | 46.2 | 1 | 0.4 | 0.4 | 141.2 | 55 |
| fentanyl | 0.809 | 0.0167 | 0.0409 | 1.37 | 2.45063 | 8.77 | 23.6 | 0 | 3.929 | 2.61 | 336.5 | 3.6 |
| flecainide | 0.846 | 0.005 | 0.0124 | 1.78 | 3.03065 | 9.3 | 59.6 | 2 | 3.466 | 1 | 414.3 | 19.5 |
| fluconazole | 1.03 | 1 | 1 | 0.57837 | 0.8706 | 1.76 | 81.7 | 1 | 0.4 | 0.4 | 306.3 | 30 |
| fluoxetine | 0.766 | 0.00158 | 0.00397 | 0.735 | 0.52066 | 9.8 | 21.3 | 1 | 4.352 | 1.41 | 309.3 | 48 |
| flurbiprofen | 0.647 | 0.00166 | 0.00066 | 1.07167 | 0.01968 | 4.22 | 37.3 | 1 | 4.115 | 0.52 | 244.3 | 5.2 |
| fluvoxamine | 0.848 | 0.0136 | 0.0335 | 0.962 | 1.28 | 8.86 | 56.8 | 1 | 3.2 | 1.26 | 318.3 | 18.5 |
| gabapentin | 1.146 | 9.99E-08 | 9.99E-08 | 1.05067 | 0.8607 | 3.7 | 63.3 | 2 | -1.1 | -4.6 | 171.2 | 6 |
| hydrochlorothiazide | 0.64 | 0.888 | 0.76 | 2.658 | 0.9906 | 7.9 | 135.1 | 4 | -0.5 | -0.5 | 297.7 | 10.5 |
| hydromorphone | 0.996 | 0.059 | 0.137 | 1.00072 | 1.8506 | 8.2 | 49.8 | 1 | 0.9 | -0.33 | 321.8 | 2.5 |
| ibuprofen | 0.754 | 0.00268 | 0.00107 | 0.70022 | 0.02573 | 4.43 | 37.3 | 1 | 3.5 | 1.4 | 206.3 | 1.9 |
| imatinib | 0.794 | 0.051 | 0.119 | 1.37 | 0.50075 | 8.27 | 86.3 | 2 | 3.5 | 2.58 | 493.6 | 18 |
| indomethacin | 0.763 | 0.00315 | 0.00126 | 1.0822 | 0.37076 | 4.5 | 68.5 | 1 | 4.3 | 1.43 | 357.8 | 4.5 |
| kanamycin | 1.863 | 0.387 | 0.613 | 0.962 | 0.22577 | 7.2 | 282.6 | 11 | -6.3 | -8.2 | 484.5 | 2.5 |
| ketorolac tromethamine | 0.756 | 0.000316 | 0.000126 | 4.30023 | 0.02578 | 3.5 | 146 | 5 | 2.28 | -1.37 | 376.4 | 2.5 |
| labetalol | 0.5 | 0.00499 | 0.0124 | 1.37 | 1.0106 | 9.3 | 95.6 | 5 | 2.7 | 0.46 | 328.4 | 5.5 |
| lamivudine | 1.121 | 0.999 | 1 | 2.778 | 1.12081 | 4.08 | 113 | 2 | -0.9 | -0.9 | 229.3 | 6 |
| lamotrigine | 0.956 | 0.952 | 0.98 | 0.4712 | 0.41383 | 5.7 | 90.7 | 2 | 1.4 | 1.39 | 256.1 | 25 |
| levetiracetam | 1.082 | 1 | 1 | 0.962 | 1.00084 | 2 | 63.4 | 1 | -0.3 | -0.3 | 170.2 | 7 |
| levodopa | 1.069 | 2.09E-05 | 8.35E-06 | 1.00085 | 0.30086 | 2.32 | 103.8 | 4 | -0.225 | -3.67 | 197.2 | 0.8 |
| levofloxacin | 1.084 | 0.00279 | 0.0025 | 3.30088 | 0.95089 | 5.5 | 73.3 | 1 | -0.4 | -2.2 | 361.4 | 7 |
| lidocaine | 0.898 | 0.089 | 0.197 | 0.80022 | 1.0709 | 8.01 | 32.3 | 1 | 2.3 | 1.6 | 234.3 | 1.8 |
| lincomycin | 0.997 | 0.137 | 0.285 | 0.962 | 0.90091 | 7.8 | 122.5 | 5 | 0.56 | -0.17 | 406.5 | 5.4 |
| loratadine | 0.68 | 0.998 | 0.999 | 1.37 | 1.20094 | 4.33 | 42.4 | 0 | 6.234 | 3.9 | 382.9 | 1.5 |
| medroxyprogesterone | 0.821 | 1 | 1 | 0.962 | 0.88095 | 17.82 | 54.4 | 1 | 3.5 | 3.5 | 344.5 | 50 |
| mefloquine | 0.75 | 0.0245 | 0.0593 | 2.20096 | 0.14597 | 8.6 | 45.2 | 2 | 3.6 | 2.38 | 378.3 | 504 |
| meperidine | 0.881 | 0.025 | 0.0606 | 1.05898 | 1.0706 | 8.59 | 29.5 | 0 | 2.6 | 1.77 | 247.3 | 3.5 |
| mepindolol | 0.908 | 0.00173 | 0.00435 | 0.962 | 0.38499 | 9.76 | 57.3 | 3 | 2.3 | 0.027 | 262.4 | 4 |
| metformin | 0.8 | 3.99E-06 | 1.00E-05 | 0.9702 | 0.4806 | 12.4 | 91.5 | 5 | -0.5 | -3.43 | 129.2 | 4 |
| methadone | 0.804 | 0.0114 | 0.028 | 1.20012 | 0.6406 | 8.25 | 20.3 | 0 | 3.93 | 1.45 | 309.4 | 35 |
| methohexital | 0.923 | 0.98 | 0.952 | 0.962 | 1.1001 | 8.7 | 66.5 | 1 | 1.8 | 1.78 | 262.3 | 3.9 |
| methotrexate | 1.01 | 0.000192 | 3.12E-05 | 1.22 | 0.0456 | 4.8 | 210.5 | 7 | -2.2 | -8.85 | 454.4 | 15 |
| methylergonovine | 0.974 | 0.112 | 0.24 | 0.962 | 0.300102 | 7.9 | 68.4 | 3 | 1.2 | 0.55 | 339.4 | 3.4 |
| methylphenidate | 1.043 | 0.008 | 0.02 | 1.78 | 1.100104 | 9.09 | 38.3 | 1 | 0.2 | -1.48 | 233.3 | 3.5 |
| metoclopramide | 0.939 | 0.00534 | 0.0133 | 1.78 | 1.900106 | 9.27 | 67.6 | 3 | 1.8 | 0.15 | 299.8 | 6 |
| metoprolol | 0.96 | 0.00199 | 0.00499 | 0.77 | 2.7906 | 9.7 | 50.7 | 2 | 1.6 | -0.63 | 267.4 | 3.5 |
| metronidazole | 1.09 | 1 | 1 | 0.693107 | 0.9106 | 2.52 | 83.9 | 1 | -0.1 | -0.1 | 171.2 | 6.5 |
| mexiletine | 0.909 | 0.00627 | 0.0156 | 0.962 | 1.450108 | 8.75 | 32.2 | 1 | 2.16 | 0.92 | 179.3 | 10 |
| midazolam | 0.786 | 0.876 | 0.947 | 1.063 | 0.09011 | 6.15 | 30.2 | 0 | 3.699 | 3.78 | 325.8 | 3 |
| minoxidil | 1.06 | 0.996 | 0.998 | 0.818111 | 0.7606 | 4.61 | 93.6 | 4 | 0.6 | 0.6 | 209.3 | 3.5 |
| mirtazapine | 0.25 | 0.166 | 0.334 | 0.962 | 0.97 | 7.7 | 19.4 | 0 | 2.9 | 2.35 | 265.4 | 30 |
| moclobemide | 0.78 | 0.905 | 0.96 | 0.962 | 0.6806 | 6.2 | 41.6 | 1 | 1.5 | 1.48 | 268.7 | 1.9 |
| morphine | 0.969 | 0.112 | 0.24 | 1.37 | 2.4606 | 7.9 | 52.9 | 2 | 1.272 | -1 | 285.3 | 3 |
| n-desmethylsertraline | 0.773 | 0.003 | 0.00753 | 1.78 | 1.64012 | 9.52 | 26 | 1 | 4.3 | 2.23 | 292.2 | 66 |
| nadolol | 0.971 | 0.00213 | 0.00534 | 1.37 | 4.600116 | 9.67 | 82 | 4 | 1.288 | -1.54 | 309.4 | 15.5 |
| naltrexone | 0.934 | 0.0124 | 0.0307 | 0.8117 | 1.900118 | 8.9 | 70 | 2 | 1.92 | 0.38 | 341.4 | 4 |
| naproxen | 0.758 | 0.00141 | 0.000562 | 1.305 | 0.16063 | 4.15 | 46.5 | 1 | 2.998 | 0.35 | 230.3 | 14.5 |
| nefazodone | 0.714 | 0.448 | 0.671 | 1.068121 | 0.270122 | 7.09 | 51.6 | 0 | 3.497 | 3.88 | 470 | 3 |
| nefopam | 0.866 | 0.16 | 0.324 | 0.962 | 1.200123 | 7.72 | 12.5 | 0 | 2.9 | 2.41 | 253.3 | 4 |
| nicardipine | 0.781 | 0.0594 | 0.137 | 1.22124 | 0.180125 | 8.2 | 113.7 | 1 | 3.82 | 2.93 | 479.5 | 8.6 |
| nicotine | 0.87 | 0.0872 | 0.194 | 1.700126 | 3.0006 | 8.02 | 16.1 | 0 | 1.1 | -0.37 | 162.2 | 15 |
| nimodipine | 0.813 | 0.975 | 0.991 | 1.78 | 0.105128 | 5.4 | 119.7 | 1 | 3.05 | 3.04 | 418.4 | 8.5 |
| nitrazepam | 0.51 | 1 | 1 | 0.962 | 0.27 | 11.9 | 87.3 | 1 | 2.25 | 2.25 | 281.3 | 24 |
| nitrendipine | 0.776 | 0.973 | 0.989 | 0.962 | 0.350129 | 5.43 | 110 | 1 | 2.9 | 2.89 | 360.4 | 16 |
| nitrofurantoin | 1.07 | 0.613 | 0.387 | 10.09137 | 6.2906 | 7.2 | 120.7 | 1 | -0.1 | -0.11 | 238.2 | 0.5 |
| norethindrone | 0.793 | 1 | 1 | 0.962 | 0.19013 | 17.59 | 37.3 | 1 | 3 | 3 | 298.4 | 9 |
| norfluoxetine | 0.797 | 0.0017 | 0.004 | 0.962 | 0.560131 | 9.77 | 35.2 | 1 | 3.5 | 1.22 | 295.3 | 240 |
| noscapine | 0.805 | 0.137 | 0.285 | 0.962 | 0.290132 | 7.8 | 75.7 | 0 | 2.7 | 2.65 | 413.4 | 2.6 |
| ofloxacin | 0.88 | 0.0383 | 0.0156 | 2.990134 | 0.9206 | 6.1 | 73.3 | 1 | 2.1 | 0.25 | 361.4 | 22.5 |
| olanzapine | 0.873 | 0.365 | 0.591 | 1.100135 | 0.2006 | 7.24 | 56.2 | 1 | 2 | 0.93 | 312.4 | 33 |
| ondansetron | 0.896 | 0.313 | 0.534 | 1.78 | 3.200137 | 7.34 | 39.8 | 0 | 2.3 | 2.03 | 293.4 | 3.8 |
| oxazepam | 0.879 | 1 | 0.999 | 0.3802 | 0.100138 | 10.61 | 61.7 | 2 | 2.2 | 2.2 | 286.7 | 15.5 |
| oxcarbazepine | 0.932 | 1 | 1 | 1.78 | 0.50014 | 13.73 | 63.4 | 1 | 1.7 | 1.7 | 252.3 | 3 |
| oxprenolol | 0.9 | 0.00213 | 0.00534 | 0.962 | 0.370141 | 9.67 | 50.7 | 2 | 2.1 | -0.098 | 265.4 | 1.5 |
| oxycodone | 1.004 | 0.0307 | 0.0736 | 1.37 | 3.200143 | 8.5 | 59 | 1 | 0.7 | -0.45 | 315.4 | 3.2 |
| pantoprazole | 0.796 | 0.938 | 0.86 | 0.962 | 0.022144 | 8.19 | 106 | 1 | 2.4 | 2.33 | 383.4 | 1.1 |
| paroxetine | 0.778 | 0.0013 | 0.0032 | 1.78 | 0.390146 | 9.9 | 39.7 | 1 | 3.89 | 1.48 | 329.3 | 21 |
| pefloxacin | 0.921 | 0.034 | 0.016 | 1.174148 | 0.960149 | 6.3 | 64.1 | 1 | 2.164 | -0.17 | 333.4 | 46.3 |
| phenacetin | 0.946 | 1 | 1 | 1.00515 | 0.670151 | 14.98 | 38.3 | 1 | 1.58 | 1.66 | 179.2 | 0.9 |
| phenytoin | 0.584 | 0.952 | 0.888 | 1.37 | 0.336153 | 8.33 | 58.2 | 2 | 2.524 | -0.71 | 252.3 | 22 |
| piroxicam | 0.727 | 0.166 | 0.074 | 0.80023 | 0.020154 | 6.3 | 108 | 2 | 3.1 | 1.96 | 331.3 | 50 |
| praziquantel | 0.44 | 1 | 1 | 1.018155 | 0.2406 | 19.38 | 40.6 | 0 | 2.5 | 2.5 | 312.4 | 1.1 |
| prednisolone | 0.86 | 1 | 1 | 1.37 | 0.1306 | 12.58 | 94.8 | 3 | 1 | 1 | 360.4 | 2.5 |
| pregabalin | 1.19 | 3.70E-06 | 3.70E-06 | 1.050157 | 1.000158 | 4.8 | 63.3 | 2 | -1.6 | -4.1 | 159.2 | 6.3 |
| primidone | 0.997 | 1 | 1 | 0.962 | 0.700159 | 11.5 | 58.2 | 2 | 0.9 | 0.9 | 218.2 | 10 |
| propranolol | 0.41 | 0.00379 | 0.00946 | 1.042 | 0.3906 | 9.42 | 41.5 | 2 | 3 | 0.78 | 259.3 | 4.5 |
| propylthiouracil | 0.31 | 0.925 | 0.83 | 0.9532 | 0.1306 | 8.09 | 73.2 | 2 | 0.4 | 0.32 | 170.2 | 1.5 |
| prucalopride | 0.928 | 0.0306 | 0.0736 | 1.78 | 2.65 | 8.5 | 76.8 | 2 | 2 | 0.87 | 367.9 | 19 |
| pseudoephedrine | 0.968 | 0.003 | 0.00753 | 0.828161 | 2.6406 | 10.25 | 32.3 | 2 | 1.4 | -0.7 | 165.2 | 6.5 |
| pyridostigmine | 0.943 | 1 | 1 | 1.78 | 0.660163 | 14 | 33.4 | 0 | 1.554 | 1.55 | 181.2 | 3 |
| pyrimethamine | 0.851 | 0.314 | 0.534 | 1.69067 | 0.56046 | 7.34 | 77.8 | 2 | 2.87 | 2.55 | 248.7 | 96 |
| quazepam | 0.779 | 1 | 1 | 0.962 | 4.180164 | 2.59 | 15.6 | 0 | 3.874 | 4.03 | 386.8 | 39 |
| quetiapine | 0.45 | 0.466 | 0.686 | 0.841165 | 0.3606 | 7.06 | 73.6 | 1 | 2.8 | 2.63 | 383.5 | 7 |
| risperidone | 0.852 | 0.017 | 0.0418 | 1.37 | 0.260167 | 8.76 | 61.9 | 0 | 2.7 | 1.33 | 410.5 | 3 |
| rofecoxib | 0.836 | 1 | 1 | 0.81723 | 0.2106 | 14.84 | 68.8 | 0 | 3.2 | 3.2 | 314.4 | 17 |
| salicylate | 0.895 | 9.33E-05 | 3.71E-05 | 0.85 | 0.050168 | 2.97 | 57.5 | 2 | 2.061 | -1.14 | 137.1 | 4 |
| sertraline | 0.709 | 0.0069 | 0.017 | 1.78 | 1.93012 | 9.16 | 12 | 1 | 4.8 | 3.05 | 306.2 | 26 |
| sotalol | 1.046 | 0.00151 | 0.00352 | 1.78 | 5.4 | 8.3 | 86.8 | 3 | 0.2 | -2.1 | 272.4 | 15 |
| sumatriptan | 1.003 | 0.00288 | 0.00719 | 1.37 | 5.5906 | 9.63 | 73.6 | 2 | 0.8 | -1.33 | 295.4 | 2 |
| suprofen | 0.863 | 0.00081 | 0.00032 | 0.962 | 0.014171 | 3.91 | 82.6 | 1 | 3.3 | -0.07 | 260.3 | 170 |
| tacrolimus | 0.831 | 0.999 | 0.997 | 1.37 | 2.2006 | 9.96 | 178.4 | 3 | 3.3 | 3.3 | 804 | 12 |
| temazepam | 0.819 | 1 | 0.999 | 0.962 | 0.140173 | 10.68 | 52.9 | 1 | 2.2 | 2.2 | 300.7 | 8 |
| terbutaline | 0.98 | 0.00171 | 0.0042 | 1.2272 | 1.5406 | 9.76 | 72.7 | 4 | 1.4 | -0.77 | 225.3 | 13.5 |
| tetracycline | 1.215 | 1 | 1 | 1.78 | 1.000175 | 3.3 | 182 | 6 | -2 | -2 | 444.4 | 8.5 |
| theobromine | 1.118 | 0.0013 | 0.0032 | 0.962 | 0.820176 | 9.9 | 67.2 | 1 | -0.8 | -3.18 | 180.2 | 3.8 |
| theophylline | 1.112 | 0.985 | 0.963 | 1.173 | 0.7506 | 8.81 | 69.3 | 1 | -0.8 | -0.8 | 180.2 | 6.5 |
| thiopental | 0.16 | 0.78 | 0.585 | 0.962 | 0.4206 | 7.55 | 90.3 | 2 | 2.3 | 1.9 | 264.3 | 10 |
| tiapamil | 0.852 | 0.154 | 0.314 | 0.962 | 0.440177 | 7.74 | 125 | 0 | 3.1 | 2.6 | 555.7 | 2.5 |
| timolol | 0.943 | 0.00627 | 0.0156 | 1.1602 | 0.800141 | 9.2 | 108 | 2 | 1.8 | 0.019 | 316.4 | 4 |
| tolmetin | 0.703 | 0.000316 | 0.000126 | 1.15523 | 0.005178 | 3.5 | 59.3 | 1 | 1.549 | -0.39 | 257.3 | 1.5 |
| topiramate | 1.119 | 0.98 | 0.952 | 1.78 | 0.90018 | 8.7 | 124 | 1 | -0.8 | -0.82 | 339.4 | 21 |
| tramadol | 0.898 | 0.00387 | 0.0096 | 1.200181 | 2.200182 | 9.41 | 32.7 | 1 | 2.6 | 0.628 | 263.4 | 5.5 |
| trazodone | 0.42 | 0.645 | 0.821 | 0.924 | 0.140183 | 6.74 | 42.4 | 0 | 2.9 | 2.73 | 371.9 | 11 |
| triprolidine | 0.46 | 0.0224 | 0.0544 | 0.962 | 0.53 | 8.64 | 16.1 | 0 | 4 | 2.74 | 278.4 | 3.2 |
| valproate | 0.865 | 0.00627 | 0.0025 | 0.962 | 0.03185 | 4.8 | 37.3 | 1 | 2.8 | 0.216 | 144.2 | 14 |
| venlafaxine | 0.881 | 0.00397 | 0.0099 | 1.37 | 2.753186 | 9.4 | 32.7 | 1 | 2.912 | 0.76 | 277.4 | 5 |
| verapamil | 0.21 | 0.0175 | 0.0428 | 1.37 | 0.8706 | 8.75 | 64 | 0 | 4.7 | 2.45 | 454.6 | 8.5 |
| verteporfin | 0.881 | 0.00132 | 0.000524 | 0.962 | 0.66 | 4.12 | 347 | 6 | 2.1 | -1.1 | 1437.6 | 5.5 |
| vigabatrin | 1.248 | 1.99E-06 | 1.98E-06 | 0.400188 | 0.430189 | 4 | 63.3 | 2 | -2.2 | -4.7 | 129.2 | 6.3 |
| zaleplon | 0.984 | 1 | 1 | 0.962 | 0.5506 | 0.3 | 74.3 | 0 | 0.9 | 0.9 | 305.3 | 3.7 |
| zidovudine | 1.053 | 0.998 | 0.995 | 1.383 | 1.45019 | 9.68 | 108.3 | 2 | 0.05 | 0.05 | 267.2 | 1.8 |
| zolpidem | 0.849 | 0.874 | 0.946 | 1.10012 | 0.130191 | 6.16 | 37.6 | 0 | 2.608 | 3.03 | 307.4 | 2.8 |
| zonisamide | 1.036 | 0.999 | 0.998 | 0.962 | 0.930192 | 10.2 | 94.6 | 1 | 0.2 | 0.2 | 212.2 | 66 |
| zopiclone | 0.996 | 0.666 | 0.834 | 0.962 | 0.51 | 6.7 | 91.8 | 0 | 0.8 | 0.45 | 388.8 | 5 |
| zuclopenthixol | 0.688 | 0.0594 | 0.137 | 0.962 | 0.290194 | 8.2 | 26.7 | 1 | 4.3 | 3.42 | 401 | 20 |

^a^, f_um_ and ER were obtained from [Yang, H., Xue, I., Gu, Q., Zou, P., Zhang, T., Lu, Y., Fisher, J., Tran, D., 2022. Developing an In Vitro to In Vivo Extrapolation (IVIVE) Model to Predict Human Milk-to-Plasma Drug Concentration Ratios. Mol. Pharm. 19, 2506–2517. https://doi.org/10.1021/acs.molpharmaceut.2c00193](https://www.zotero.org/google-docs/?cor5al).

^b^, F_ni7.0_ and F_ni7.4_ were predicted using SimCYP (Certara USA Inc., Princeton, NJ) prediction toolbox.

^c^, The M/P ratio was calculated as the ratio of milk AUC to plasma AUC from the exposure data obtained from literature (Yang et al., 2022).

^d^, MW, HBD, logP, pKa, PSA, and T_1/2_ were obtained from the drugbank website (<https://go.drugbank.com/>) or drug label (FDA).

^e^, The logD values were estimated based on the equations of calculating logD from logP as described in Methods.

**Supplementary Table 2: Summary of prediction results from all methods with each cross-validation fold as testing data.**

| **Classification** | **Method** | **Average Accuracy** | **SD of Accuracy** | **F-1 score** | **Fold** | **Accuracy in each fold** | **F-1 score in each fold** |
| --- | --- | --- | --- | --- | --- | --- | --- |
| Two Categories | KNN | 78% | 0.04 | 0.70 | 1 | 0.77 | 0.70 |
|  |  |  |  |  | 2 | 0.73 | 0.63 |
|  |  |  |  |  | 3 | 0.77 | 0.70 |
|  |  |  |  |  | 4 | 0.85 | 0.80 |
|  |  |  |  |  | 5 | 0.76 | 0.63 |
|  | RF | 78% | 0.07 | 0.69 | 1 | 0.73 | 0.63 |
|  |  |  |  |  | 2 | 0.77 | 0.57 |
|  |  |  |  |  | 3 | 0.92 | 0.89 |
|  |  |  |  |  | 4 | 0.73 | 0.67 |
|  |  |  |  |  | 5 | 0.76 | 0.67 |
|  | SVM | 78% | 0.03 | 0.71 | 1 | 0.73 | 0.67 |
|  |  |  |  |  | 2 | 0.77 | 0.70 |
|  |  |  |  |  | 3 | 0.77 | 0.70 |
|  |  |  |  |  | 4 | 0.81 | 0.76 |
|  |  |  |  |  | 5 | 0.8 | 0.74 |
|  | NN | 82% | 0.09 | 0.82 | 1 | 0.68 | 0.72 |
|  |  |  |  |  | 2 | 0.90 | 0.96 |
|  |  |  |  |  | 3 | 0.84 | 0.78 |
|  |  |  |  |  | 4 | 0.75 | 0.93 |
|  |  |  |  |  | 5 | 0.93 | 0.71 |
| Three Categories | KNN | 60% | 0.06 | 0.57 | 1 | 0.61 | 0.55 |
|  |  |  |  |  | 2 | 0.65 | 0.63 |
|  |  |  |  |  | 3 | 0.62 | 0.57 |
|  |  |  |  |  | 4 | 0.65 | 0.65 |
|  |  |  |  |  | 5 | 0.48 | 0.46 |
|  | RF | 64% | 0.07 | 0.62 | 1 | 0.69 | 0.64 |
|  |  |  |  |  | 2 | 0.50 | 0.49 |
|  |  |  |  |  | 3 | 0.65 | 0.65 |
|  |  |  |  |  | 4 | 0.65 | 0.66 |
|  |  |  |  |  | 5 | 0.68 | 0.63 |
|  | SVM | 63% | 0.02 | 0.60 | 1 | 0.62 | 0.55 |
|  |  |  |  |  | 2 | 0.65 | 0.62 |
|  |  |  |  |  | 3 | 0.65 | 0.63 |
|  |  |  |  |  | 4 | 0.62 | 0.62 |
|  |  |  |  |  | 5 | 0.60 | 0.59 |
|  | NN | 68% | 0.12 | 0.61 | 1 | 0.48 | 0.47 |
|  |  |  |  |  | 2 | 0.65 | 0.72 |
|  |  |  |  |  | 3 | 0.77 | 0.69 |
|  |  |  |  |  | 4 | 0.84 | 0.57 |
|  |  |  |  |  | 5 | 0.66 | 0.61 |

**Figure 1:** Plot of PCA analysis by M/P ratio categories. A, PC1 against PC2 from PCA analysis for two categories. Blue, M/P ratio of [0, 1]; Orange, M/P ratio of [1, 8]. B, PC1 against PC2 from PCA analysis for three categories. Blue, M/P ratio of [0, .5]; Orange, M/P ratio of [0.5, 1]; Green, M/P ratio of [1,8].

**A.**


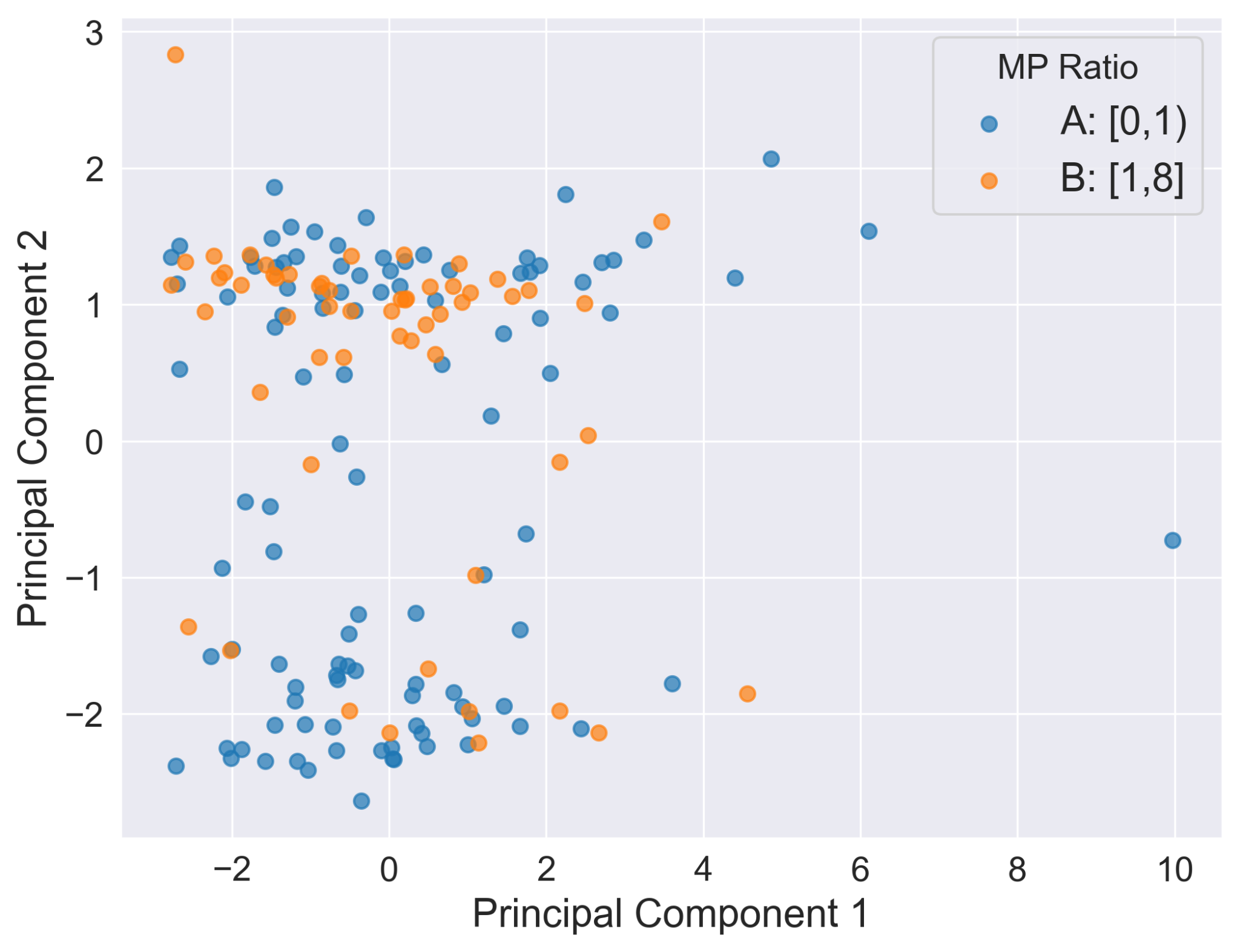


**B.**


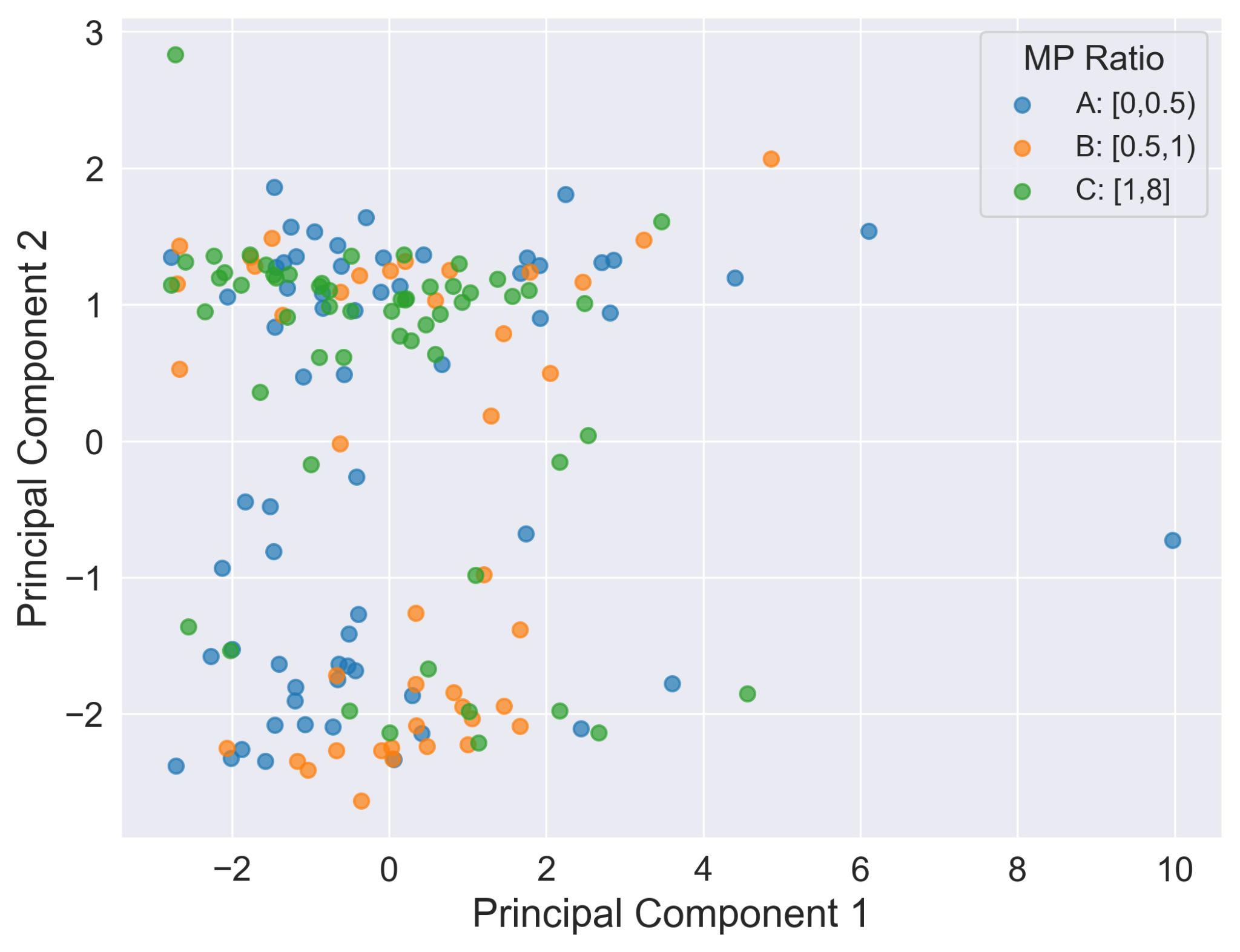


**Figure 2:** Feature importance ranking for Random Forest. A, Bar plot for feature importance from high to low value for two categories Random Forest. B, Bar plot for feature importance from high to low value for three categories Random Forest.

A.


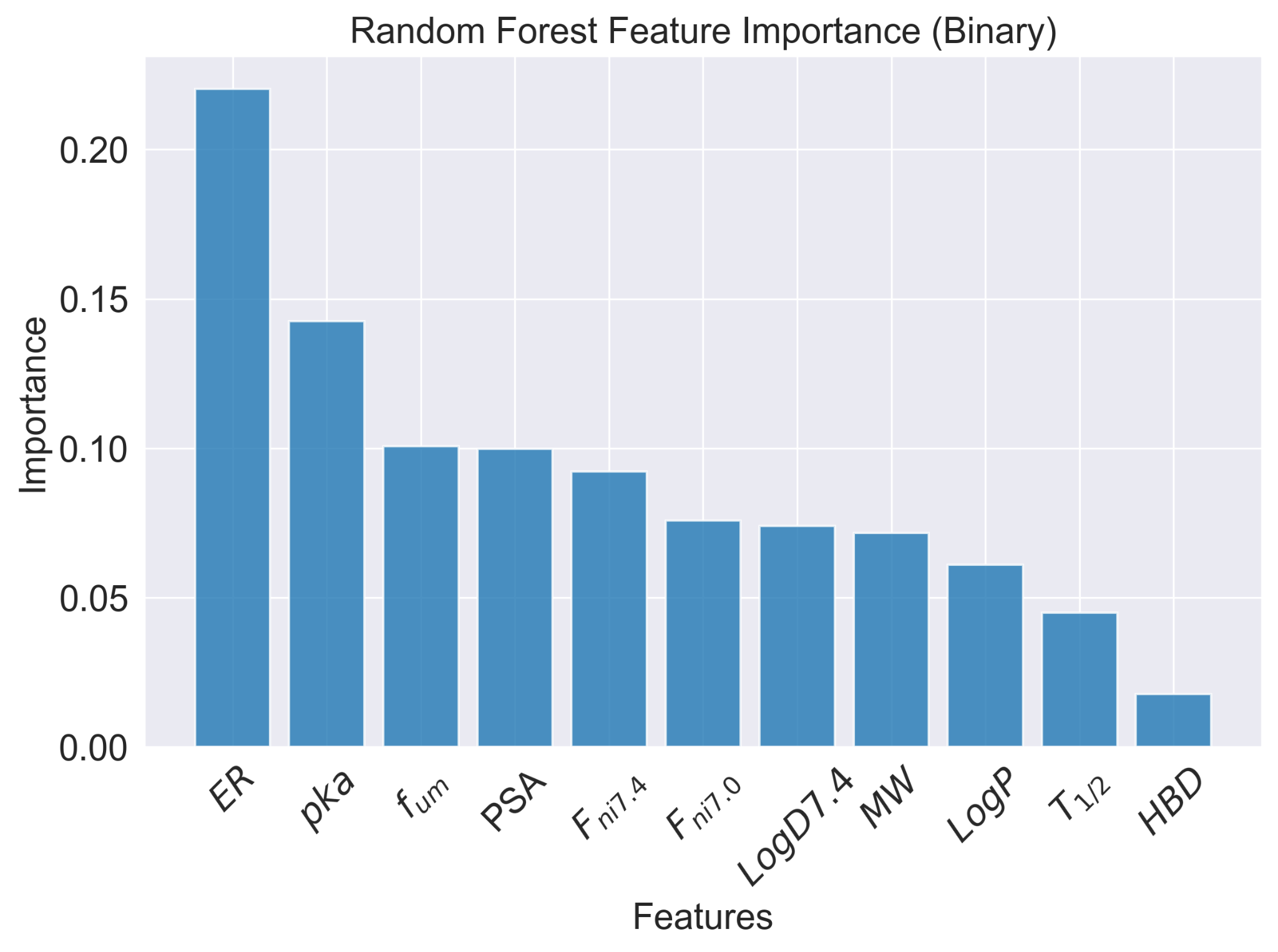


B.


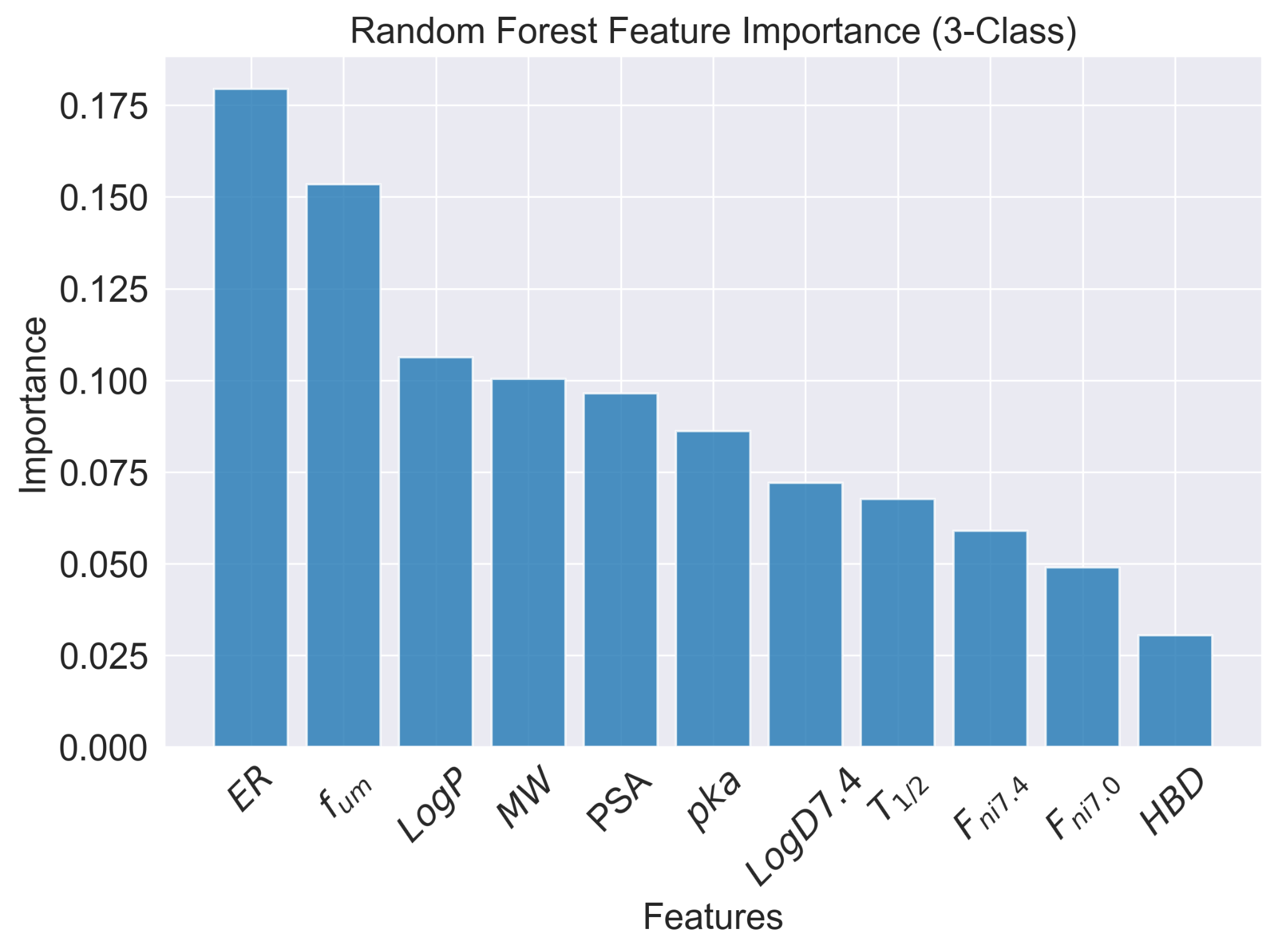


**Figure 3:** Feature importance ranking for Support Vector Machine. A, Bar plot for feature importance from high to low value for two categories Support Vector Machine. B, Bar plot for feature importance from high to low value for three categories Support Vector Machine.

A.


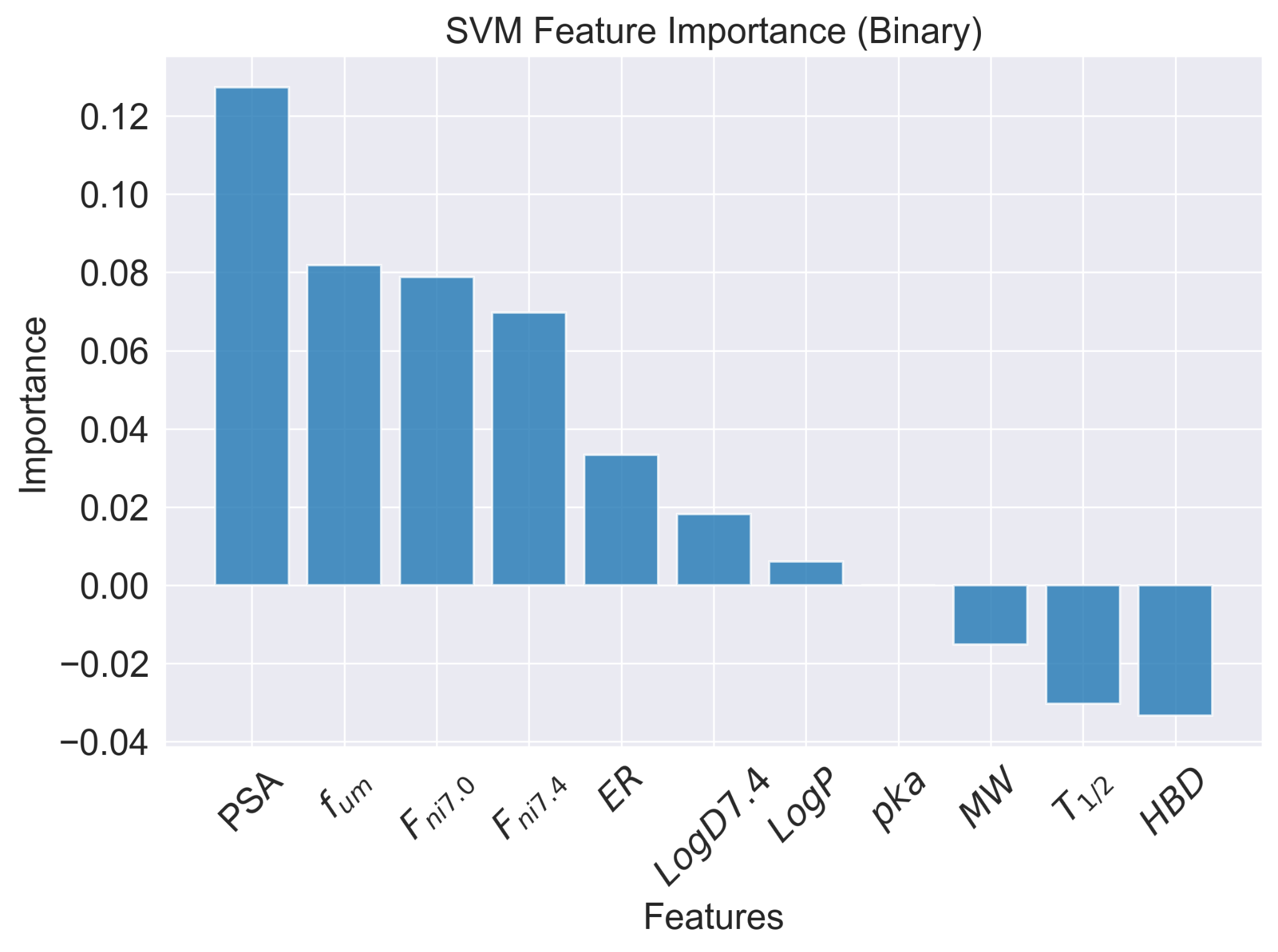


B.


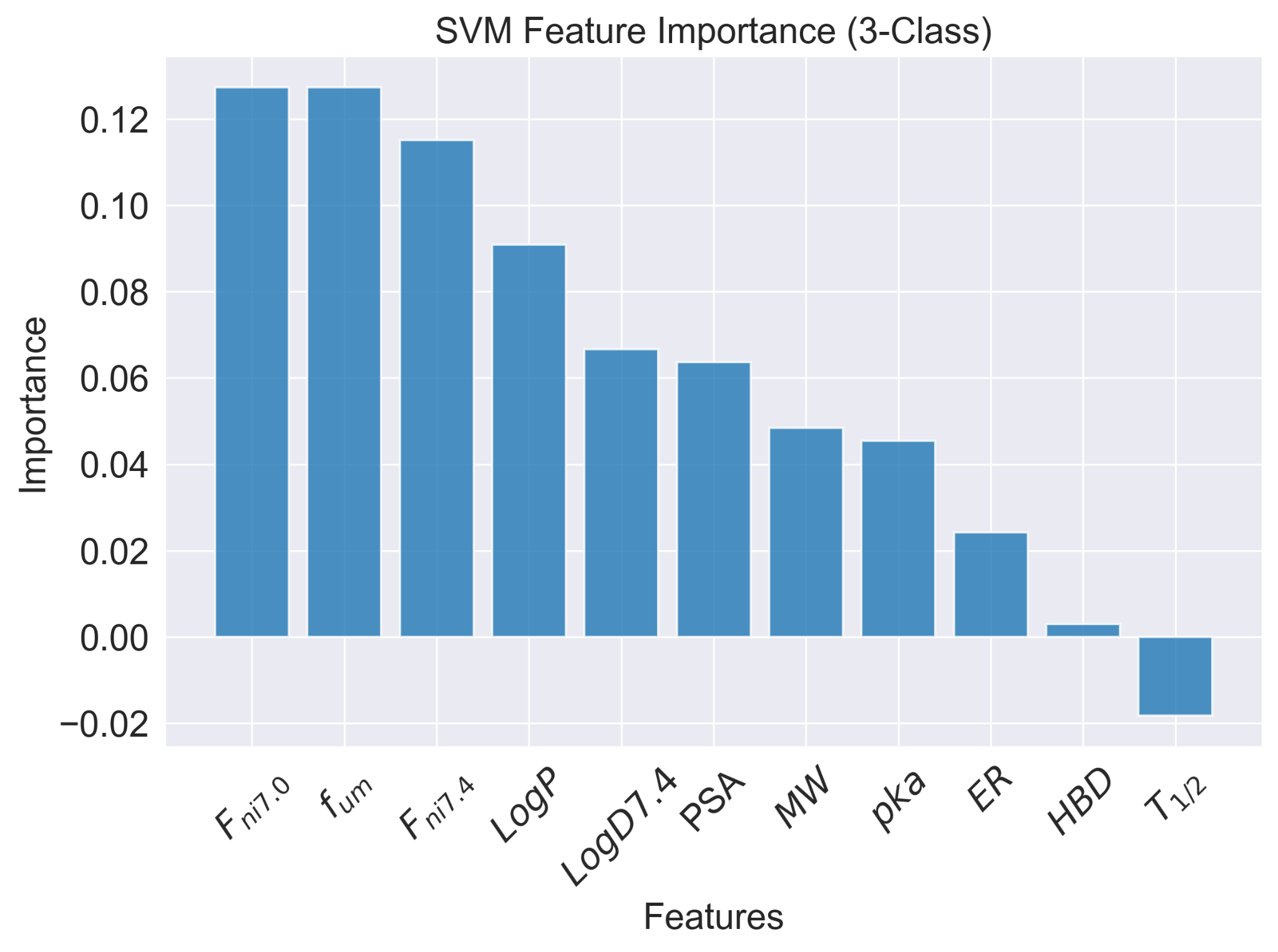


**Figure 4:** Feature importance ranking for Neural Network. A, Bar plot for feature importance from high to low value for two categories Neural Network. B, Bar plot for feature importance from high to low value for three categories Neural Network.

A.


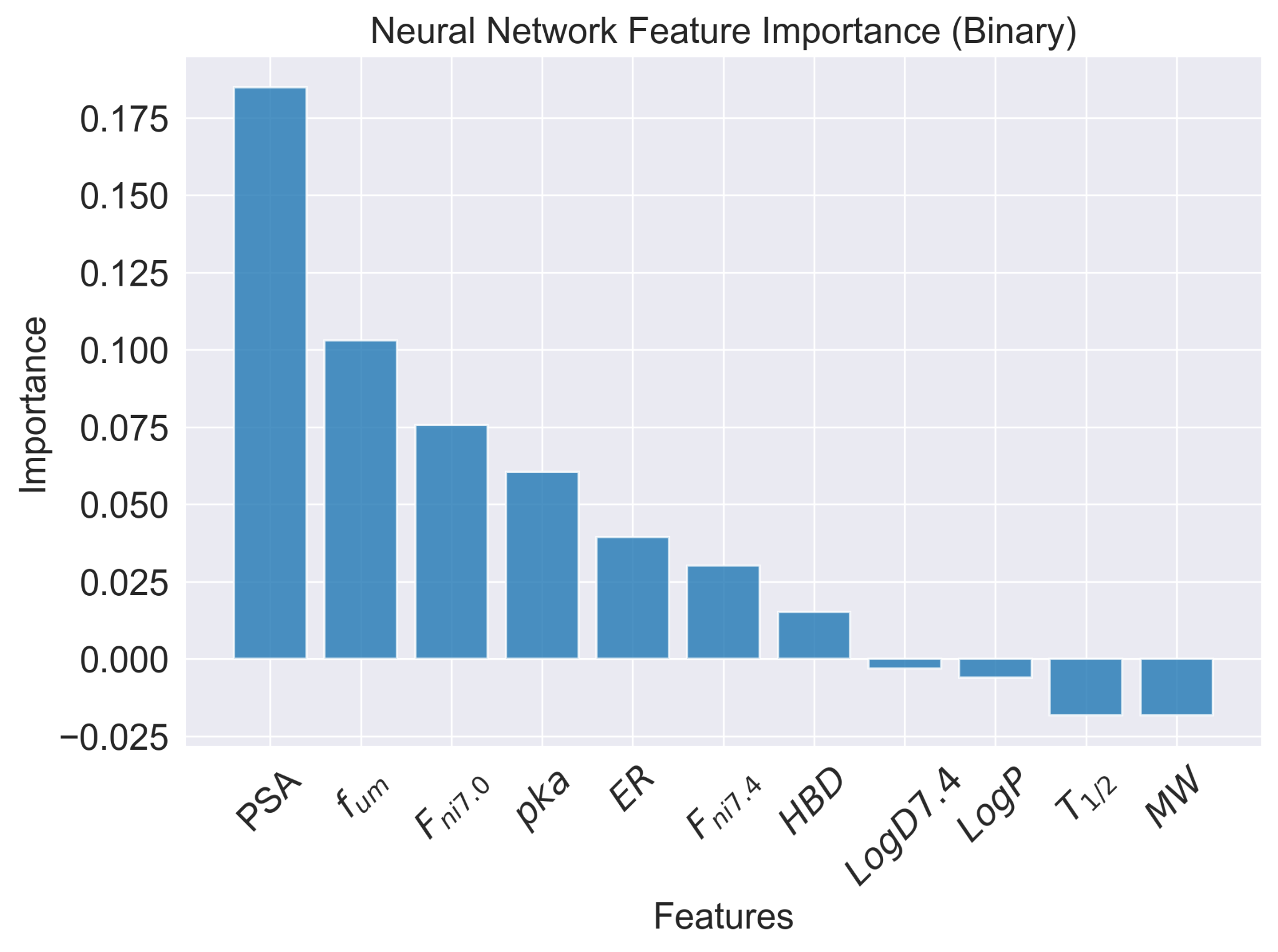


B.


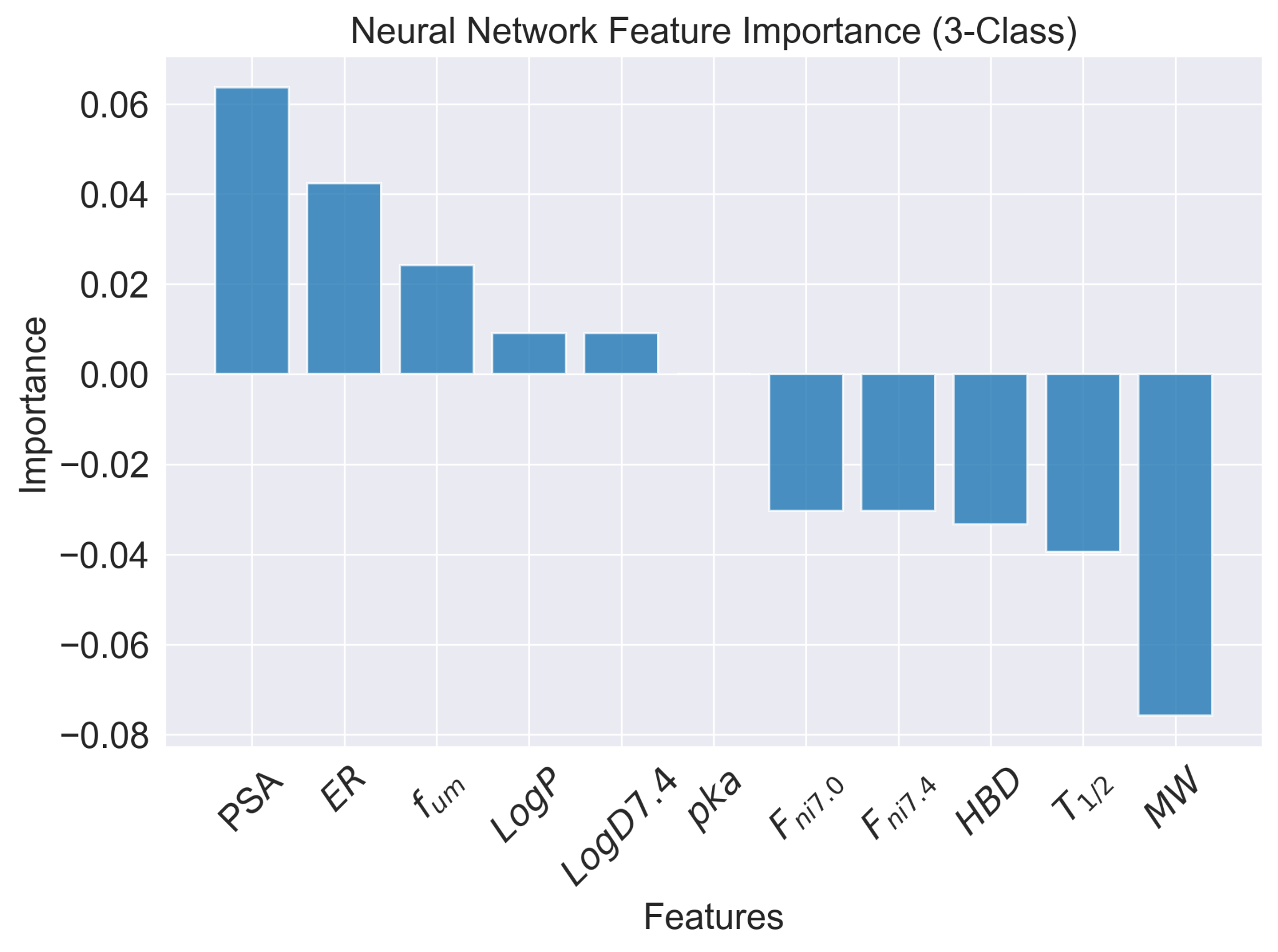

Supplement: Supplementary file 1 — Supplementary file1 (DOCX 2632 KB) [file 10928_2025_9972_MOESM1_ESM.docx]
